# Study protocol for a non-randomized controlled trial of the effects of internet-based parent training as a booster to the preschool edition of PATHS®: Universal edition of the Parent Web

**DOI:** 10.1371/journal.pone.0284926

**Published:** 2023-04-27

**Authors:** Tina M. Olsson, Pia Enebrink, Sabina Kapetanovic, Laura Ferrer-Wreder, Johanna Stålnacke, Lilianne Eninger, Kyle Eichas, Åsa Norman, Lene Lindberg, Ingela Clausén Gull, Hanna Ginner Hau, Mara Westling Allodi, Mina Sedem

**Affiliations:** 1 School of Health and Welfare, Jönköping University, Jönköping, Sweden; 2 Department of Social Work, University of Gothenburg, Gothenburg, Sweden; 3 Division of Psychology, Department of Clinical Neuroscience, Karolinska Institute, Solna, Sweden; 4 Department of Psychology, Stockholm University, Stockholm, Sweden; 5 Department of Behavioral Studies, University West, Trollhättan, Sweden; 6 Department of Psychological Sciences, Tarleton State University, Stephenville, TX, United States of America; 7 Department of Global Public Health, Karolinska Institute, Solna, Sweden; 8 Center for Epidemiology and Community Medicine, Region Stockholm, Stockholm, Sweden; 9 Department of Special Education, Stockholm University, Stockholm, Sweden; Public Library of Science, UNITED STATES

## Abstract

Well implemented, universal parental support is often effective in families with younger children, but research on their effects on families with adolescent children is scarce. In this study, a trial of the universal parent training intervention “Parent Web” in early adolescence is added to the social emotional learning intervention Promoting Alternative Thinking Strategies (PATHS®), completed in early childhood. The Parent Web is a universal online parenting intervention based on social learning theory. The intervention aims to promote positive parenting and family interaction through five weekly modules completed over 6–8 weeks. The main hypothesis is that participants in the intervention group will exhibit significant pre- to post- intervention-related benefits relative participants in the comparison group. The aims of this study are: 1) provide Parent Web as a booster aimed at improving parenting support and practices at the transition into adolescence to a cohort of parents whose children have previously participated in preschool PATHS, and 2) examine the effects of the universal edition of Parent Web. The study has a quasi-experimental design with pre- and post-testing. The incremental effects of this internet-delivered parent training intervention are tested in parents of early adolescents (11–13 years) who participated in PATHS when 4–5 years old compared to a matched sample of adolescents with no prior experience of PATHS. The primary outcomes are parent reported child behavior and family relationships. Secondary outcomes include self-reported parent health and stress. The proposed study is one of the few trials to test the effects of universal parental support in families of early adolescents and will therefore contribute to the understanding of how mental health in children and young people can be promoted across developmental periods through a continuum of universal measures.

**Trial registration:** Clinical trials.gov (NCT05172297), prospectively registered on December 29, 2021.

## Introduction

This study is complex in that it adds a trial of a universal parent training intervention (i.e., the Parent Web, PW) in early adolescence to a completed trial of a social emotional learning (SEL) intervention called preschool PATHS [Promoting Alternative Thinking Strategies; 1] which was implemented with a cohort of Swedish children during early childhood (4–5 years old) in their preschools [2, 3]. SEL encompasses an understanding of the personal and social capabilities that work to produce adequate to optimal interpersonal functioning in developmentally important contexts such as in school [4]. Universal school-based SEL interventions have, in meta-analytic reviews, demonstrated significant intervention-related benefits on participants’ social and emotional skills, attitudes, behavior, and academic performance [5, 6].

The preschool edition of PATHS is designed to promote social emotional competence [1]. Social emotional competence (SEC) involves the integration of cognitive, affective, and behavioral skills and competencies within oneself as well as in social contexts with important others [7]. SEC sets off a cascade of experiences and learning that propels the individual child’s development forward by way of learning from relationships, as well as gaining insight into one’s own emotions and capabilities to affect change in the world [7]. SEC is important throughout the lifespan and can be evidenced by children’s experience and management of their own emotions, as well as shown when children engage with others in ways that move their own development forward and open the environment for children to explore and learn [8, 9]. SEC in childhood is a powerful predictor for a range of positive developmental outcomes in adolescence and adulthood [10, 11].

SEC is also an important construct because it is foundational to the completed preschool PATHS trial [2] on which the current study builds and advances. Similarly, from a conceptual standpoint, SEC is also relevant for the Parent Web (PW) in that PW emphasizes increasing validation of children’s emotions by parents and boosting the use of warm/supportive parenting practices. These parenting practices are intended to foster adolescents’ SEC and well-being, as well as improve the overall emotional climate within the family. As such, PW shares some important intervention goals and content focus with school-based interventions such as PATHS. Unique to PW, however, is its greater emphasis on having a cognitive behavioral basis in relation to parenting and family life.

The aim of this study is twofold: 1) to provide a booster intervention (namely PW) aimed at improved parenting support and practices at the transition into adolescence to a cohort of parents whose children have previously participated in preschool PATHS, and 2) to examine the (incremental) effects of the universal edition of PW in a quasi-experimental trial.

### The Swedish PATHS® project

The Swedish PATHS trial was a cluster randomized trial conducted between April 2014 and June 2016 [2]. Briefly, the preschool edition of PATHS is a school-based, teacher implemented universal SEL intervention developed in the U.S. and designed to promote SEC in children as a foundation for improved mental health, academic success, and overall development [1]. PATHS is delivered as a curriculum by teachers and is developmentally focused on creating a caring and emotionally responsive school and classroom climate, and is grounded in theories about brain and social emotional development in children [1]. The PATHS trial assessed the short-term (pre- to posttest) effects of PATHS in an urban Swedish preschool setting. Participating children were four to five years old (N = 285; 140 PATHS intervention group; 145 wait-list control group) and were included into the cluster (k = 26 preschools; 13 interventions; 13 wait-list control) randomized trial in two waves. Over the academic school year, children that participated in PATHS evidenced improvements in working memory and prosocial play but also showed higher occurrence of teacher-rated hyperactive behaviors compared to children in the control group. Additionally, girls in the PATHS group showed improvements in emotional knowledge and reduced anxiety relative girls in the control group [2]. Other research has examined PATHS- related outcomes in the original trial in terms of outcome moderation by income level of neighborhoods in which preschools were located [12].

In the upcoming study, the participants are the parents of the PATHS cohort children and will be recruited to the immediate intervention condition of the PW trial, with the goal to examine the effects of a universal edition of PW. The wider motivation for the upcoming study is to increase our understanding of how universally provided SEL during early childhood can be coupled with universally provided parent training programs in early adolescence to support families with children and adolescents across important developmental and transitional periods (i.e., from early childhood through early adolescence).

### Parent training interventions

PW is rooted in wider research and theoretical literature on parenting interventions. Such interventions span from promotion, through prevention and treatment [13–16]. The level of intervention is often geared towards the presence and severity of problems (i.e., internalizing and externalizing) that children and/or adolescents present, and parents’ exposure to risk including social disadvantage [16, 17]. Across intervention level, parent training interventions, when well implemented and able to reach parents, have been associated with significant benefits for parents and children. These benefits include positive changes in parenting skill and reduced child behavioral problems [13, 15, 17–20]. Parents of adolescents as well as parents with access to fewer social and material resources generally are yet to benefit as much from evidence-based parent training interventions, relative to parents of young children and parents with more resources [16, 17]. Additionally, in the wider parenting intervention field, there is more knowledge about beneficial interventions when children are showing heightened problems [i.e., at the selected, indicated and treatment levels of interventions; e.g., 15, 16] and less is known about how parenting programs may provide universal benefit.

Thus, the universal edition of PW has the potential to fill an important gap in our scientific understanding of how universally provided parent training may benefit children, families, and society. In addition, it is consistent with the trend to improve health and well-being equity (i.e., program reach) through the digitalization of interventions which is supported by a growing evidence that parenting interventions can be successfully delivered through online and other digital platforms [17, 21].

Indeed, the developmental timing (early adolescence) of providing PW in this study may also provide benefit as this developmental phase can present challenges to families and adolescents. Mental ill health and social adjustment problems become increasingly common during this developmental period for certain groups of youth [22–25] despite this period being characterized by thriving and developmental accomplishment for many adolescents [25, 26]. This period is also characterized by a possible general realignment of the parent-child relationship, increased romantic relationships and peer/friendship changes. There are also contextual transitions occurring during this period, as young people move through different school contexts (primary to secondary education) and may experience increasingly higher expectations for academic achievement [27]. Thus, there is past support for offering multi-focus and multi-developmental period interventions [28] in order to buffer against the consequences of these challenges which informs our working hypothesis for the upcoming study. As such, offering a universal parent training intervention at the beginning of adolescence (11 to 13 years old) as a booster to parents of children who participated in a preschool SEL intervention could improve adolescent and family well-being. This goal would be accomplished by providing a continuum of universal support in a diverse cohort of youth and their parents across developmental periods. In addition, PW may be beneficial and of broad interest to a diversity of families, and as such is likely to fill key gaps in the parenting intervention research literature (i.e., a universal intervention for parents of adolescents that is delivered online).

### WHO trial registration data set

Trial registration: clinical trials.gov (NCT05172297), https://www.clinicaltrials.gov/ct2/show/NCT05172297

Date of registration: prospectively registered on December 29, 2021.

Primary sponsor: Stockholm University

Contact for public and scientific queries: PATHS_PW.Project@psychology.su.se.

## Materials and methods

### Aim, design, and setting

The proposed study aims to develop our understanding of how mental health in children and young people can be promoted across developmental periods through a continuum of universal measures. This study will be carried out as a non-randomized controlled experiment (i.e., quasi-experiment) with a matched wait-list control condition and with dependent pre- and post-tests [29]. The study examines the incremental effects of an internet-delivered parent training for parents of early adolescents (11–13 years old) who participated in a preschool implemented social-emotional learning (SEL) intervention (PATHS) when 4–5 years old (i.e., intervention group) compared to a matched sample (i.e., comparison group).The main hypothesis is that participants in the intervention group will exhibit significant pre- to posttest intervention related benefits relative participants in the comparison group on several key outcomes.

### Target population and sample size

The target population for this study is the parent(s) of the 144 children (now 11–13 years old) that participated in the Swedish PATHS project (i.e., immediate intervention group) along with the parent(s) of a matched sample of 144 children (i.e., comparison group) for a total sample size of 288 families. Inclusion criteria is that the child took part in the Swedish PATHS intervention trial and their parents consented to have future contact with the PATHS research group and that the child and participating parents presently live in Sweden.

### Matching procedure

The recruitment pool of families will be identified via Statistics Sweden’s national registry. Matching will be made on the following variables: PATHS child’s gender, age (birthdates within 6 months), and living in the same postal code and/or municipality as the primary residence of the matched PATHS child. Statistics Sweden will identify a matched recruitment pool of families and provide contact information for the matched families to the research team (parents’ and matched child’s name, phone number, matched child’s home address). The research team is responsible for recruitment of all study participants.

### Recruitment

The first contact with the families will be by written letter to home addresses via regular post, subsequent contacts in the case of non-response will be by telephone or text message. If families consent to participate in the study, we will ask parents to update contact information for the family: parents’ and child’s name, home addresses, and phone numbers of parents/current legal guardians. This information is recorded on the consent signature form for parents stored electronically in an encrypted cloud service or on password-protected computers in a local, closed computer system at the Department of Psychology.

### Measurement

Measurement will occur at three (i.e., T1, T2, T3) time periods (See Table 1 Schedule of enrollment, interventions, and assessments). First, pre-test measurement (T1) will occur directly prior to participants’ engagement in the internet-delivered parent training program but following their informed consent. Second, posttest measurements (T2) will be taken following completion of the internet-delivered training program at approximately 6 to 8-weeks following pre-measurement. T3 will only occur for the comparison condition parents who after the wait-list time period will have completed the Parent Web.

**Table 1:** Schedule of enrollment, interventions, and assessments.

			0	T1		T2		T3
Activity/assessment	Staff Member roll	Approximate time to complete	Pre-study consent, all groups	Pre-test Baseline, all groups	Parent Web, PW group 6–7 weeks	Post-test, all groups 6-8weeks	Parent Web, control group 12-15weeks	Follow-up, control group 12-16weeks
Consent			x					
Access to web-portal				x				
Demographics, background information				x				
COVID-parent				x				
SDQ				x		x		x
DBD-OD				x		x		x
FCU				x		x		x
PARCA				x		x		x
PSS				x		x		x
HAD				x		x		x
ASQ				x		x		x
Fidelity measures				x		x		x

#### Information and data collected at pre-test only

*Parent reported background information*. Background information is collected at pre-test measurement and includes the following variables: Parent gender, age, monthly household income, education level, language(s) spoken in the home, and educational status for the index child [9 questions; see 30].

*Mental health and wellbeing during COVID–parent (CP)*. The proposed study will be conducted in temporal proximity to the COVID pandemic. Understanding how this may impact on study outcomes is an important historical factor. The COVID-parent instrument is a 10-item measure which investigates the mental health and wellbeing of respondents specifically related to the COVID pandemic [31]. The measure has been found to have good internal consistency in prior studies [Cronbach’s alpha .82; 32].

#### Primary outcomes—parent reported measures at pre- and posttest

*Strengths and Difficulties Questionnaire (SDQ)–parent ratings of child mental health/wellbeing*. The parent version of the SDQ [33, 34] is a 25-item measure for parents of children aged 2–17. Each question is answered on a three-point scale with the response options "not true", “somewhat true" and " certainly true" [34, 35]. The SDQ has shown good reliability in Swedish parent samples, ranging from .85 to .91 [36].

*Disruptive Behavior Disorder scale–oppositional defiant subscale (DBD–OD)*. The DBD oppositional defiant subscale consists of seven items related to the parent’s view of their child’s behavioral problems and defiant behavior [37]. DBD questions are rated on a 4-point scale ranging from 1 (not at all) to 4 (very much). The instrument has shown good reliability in prior studies of youth [Cronbach’s alpha .86 - .90; 30].

*Adult-Child Relationship Scale–Family Check-Up (FCU) Caregiver Assessment*. The FCU measures warmth and conflict with eight items rated on a five-point scale [30, 38]. Scale alphas in previous studies have shown good internal consistency [Cronbach’s alpha .74 - .85; 30].

#### Secondary outcomes—parent reported measures at pre- and posttest

*Parenting Children and Adolescents Scale (PARCA)*. The PARCA is a 19-item measure with each item rated on a five-point scale. The instrument includes three subscales: encouragement of positive behaviors, setting limits, and proactive parenting behaviors and has shown good psychometric properties [Cronbach’s alpha .86 - .91; 39].

*Perceived Stress Scale (PSS)*. The PSS [40] is a frequently used instrument with 10 questions about perceived stress during the last month rated on a five-point scale. The PSS measures the extent to which situations in one’s life are perceived as stressful as well as current stress levels. The instrument has shown good internal consistency [Cronbach’s alpha .80 - .89; 30].

*Hospital Anxiety and Depression Scale (HAD)*. The HAD measures the prevalence of depression and anxiety on a four-point scale. The HAD consists of 14-items and two subscales (i.e., anxiety, depression). The instrument is well validated and has been used in large studies both in Sweden and internationally [Cronbach’s alpha .70 - .81; 30].

*Affective Style Questionnaire–Tolerating subscale (ASQ)*. The Tolerating subscale from the ASQ [41] consists of five items that examine to what extent individuals experience and manage arousing emotional experiences in a non-defensive and comfortable way. The scale is answered on a five-point scale. Internal consistency was good in a prior Swedish study [Cronbach’s alpha .80 - .89; 30].

#### Implementation

Implementation of the Parent Web will be monitored through embedded measurement of parents’ satisfaction with the Parent Web as well as system monitoring of lesson *coverage* and time spent in modules as in the selective Parent Web intervention [30].

### Translation

Several of the scales were already available in Swedish (i.e., CP, SDQ, PSS, HAD) with translation conducted by other research teams and other scales (e.g., PARCA) were translated from English to Swedish with forward and back translation by qualified researchers on our research team.

### Interventions

#### Internet-delivered parent training (Parent Web)

The Parent Web is an internet-delivered parent training program consisting of five main modules: an introductory and goal-setting module and four working modules targeting: 1) Increasing the time spent with your adolescent, 2) Listening to and validating your adolescent, 3) Choosing your battles and using problem solving techniques, and 4) Balancing your adolescent’s need for independence while still being responsible for her/his well-being. Additionally, there are six optional bonus modules about concerns parents might have (youth psychological ill-health, household responsibilities, school-related problems, friends and bullying, internet and Internet-based games, alcohol, and drugs). The delivery of the bonus sections is based on specific family need and preferences. The modules are ordered such that the priority is to facilitate a trusting and caring parent-adolescent relationship. In later modules, skills for improved handling of conflicts and adolescent independent behaviors are introduced. Each module encloses facts in written text, illustrations, movie clips with interviews, lectures, or role-played parent-adolescent conflict/modeled situations and includes exercises and homework expected to take about 1 hour to complete. Bonus sections are shorter and focus on information and strategies for handling specifically challenging situations. Each parent is assigned a “family guide” who communicates through the web-portal and provides reminders about working through the modules, short feedback on homework/exercises, and answers questions posed by parents weekly. Parents are required to be active by for example answering questions, reflecting on information/video-clips, and completing homework to access the next module. The program takes on average 6–7 weeks to complete depending on the time needed for each module and the number of bonus sections completed. The program is based on social learning- and developmental theory and the parenting skills provided are in line with those learned in cognitive behavior therapy parent training programs.

#### Control

The control group will be placed on a waitlist for the Parent Web which they will be able to participate in following post-intervention data collection (i.e., after 6 to 8-weeks). No additional services will be provided to the control group during the wait-list period.

### Data collection and management plan

The Parent Web is accessed through an encrypted web-portal with double authorization. After parents (both treatment and control groups) provide online consent to participate in PW they will be contacted by the research team who will provide the parents with access information for the web-portal. The primary contact with the parents will be by telephone and subsequently through texts or web-portal notifications. Due to variation in study inclusion dates, parents enter the study on a rolling basis.

#### Parent-web (treatment) group

Following login to the portal, parents are asked to fill in the T1-survey. Parents in the Parent-web (treatment) group gain access to the PW following T1 data collection. As they complete sessions, parents are invited to a new session of PW. When finished they will be asked to fill in the new survey (T2) via the web-portal.

#### Control group

Following login to the portal, parents are asked to fill in the T1-survey. Approximately 6–8 weeks later, when treatment group parents have completed their participation in PW, parents in the control group are invited to complete T2 data collection. Parents in the control group gain access to the PW following T2 data collection. When they are finished, they will be invited to a new survey (T3) via the web-portal.

### Data analysis plan

The main hypothesis that participants in the Parent Web condition will exhibit intervention-related pre- to posttest benefits relative to participants in the comparison group will be evaluated using two-wave two-group latent curve models [42] for each of the outcome variables. The pretest level of each outcome will be modeled as a latent intercept, and pre- to posttest change in the outcome will be modeled as a latent slope. Group 1 will be the intervention group comprised of participants in Parent Web. Group 2 will be the matched wait-list control comparison group.

#### Model specification

For each outcome variable, four two-group models reflecting four alternative hypotheses will be specified and compared. First, Model A will include for both groups a latent intercept but not a latent slope (i.e., a latent change factor), reflecting the hypothesis that scores on the outcome variable will not change from pre- to posttest for either the intervention group or the matched comparison group. Second, Model B will include a latent slope for the intervention group but not for the matched comparison group, reflecting the hypothesis that scores for the intervention group will change, but scores for the matched comparison group will not change. Third, Model C will include a latent slope for the matched comparison group but not for the intervention group, reflecting the hypothesis that scores for the matched comparison group will change, but scores for the intervention group will not change. Fourth, Model D will include latent slopes for both groups, reflecting the hypothesis that scores for both the intervention group and the matched comparison group will change. For each model, outcome variables will be modeled as latent variables with multiple indicators (either items or item parcels) to account for measurement error. Thus, latent intercepts and latent slopes will be second-order factors.

#### Model estimation and model testing

All models will be estimated in Mplus 8.4 [43] using a robust maximum likelihood estimator and a Huber-White adjustment to the standard errors and chi square test of model fit, as implemented in Mplus’s TYPE = COMPLEX feature, to consider cluster sampling of participants within schools. Each model will be assessed with the chi square test of exact fit and its *p*-value, the comparative fit index (CFI), the Tucker-Lewis index (TLI), the root mean square error of approximation (RMSEA), and the standardized root mean square residual (SRMR). Good model fit will be indicated by: chi square *p*-value > .05, CFI > .95, TLI > .95, RMSEA < .06, and SRMR < .08 [44].

The main hypothesis will be evaluated for each outcome variable by comparing Models A-D using the Bayesian information criterion (BIC). When comparing models, lower BIC indicates better relative model fit; a BIC difference of greater than 10 indicates very strong evidence against the model with the largest BIC [45]. If Model D is selected, then the difference in change between the groups will be evaluated by constraining the mean and variance of the latent slope to be equal across groups and comparing this model with an unconstrained model using the Satorra-Bentler nested chi-square test for robust maximum likelihood. In addition, the sensitivity of each selected model to the assumption of pretest equivalence between groups will be tested by constraining the mean and variance of the latent intercept to be equal across groups and comparing this model with an unconstrained model, as suggested by [42].

To assess power for this analysis, we conducted a Monte Carlo simulation using Model D. The model included two groups (intervention and comparison), latent intercepts and slopes for both groups, and three continuous indicators (e.g., parcels) at both pretest and posttest. We specified population values of .70 for all standardized factor loadings and a mean difference of .50 between the latent slopes of the two groups. We assumed a sample size of 288, with 144 in each group. Under these assumptions and using 1,000 replications and a robust maximum likelihood estimator, the power estimate for the group difference in latent slopes was .88.

### Ethical considerations and declarations

The study will be based on data collected for a study that was granted ethical approval on November 15, 2021 by the Swedish Ethical Review Authority (Dnr 2021–04552), addendums for the ethics approval were approved on May 20, 2022 (Dnr 2022-02454-02) and November 23, 2022 (Dnr 2022-05932-02). Informed consent will be obtained from all individual participants included in the study. The current study will be performed in line with the principles of the Declaration of Helsinki and its later amendments, as well as GDPR ethical rules. Ethical approval will be sought for any substantive changes to the protocol.

### The status and timeline of the study

Data collection has begun in Fall 2022 and is ongoing.

## Discussion

This study will offer understanding of how interventions can promote children’s mental health when delivered at different developmental stages. Findings will increase our understanding of the extent to which a SEL program delivered by teachers in preschool may have long lasting effects on adolescents’ mental health. Further, results will provide knowledge regarding the possible booster effects of an online parenting program (PW) on adolescents’ mental health. Considering the high numbers of children and adolescents with mental health problems and disorders [46, 47], especially during COVID-19 [48] it is of value to assess the usefulness of tools for mental health promotion as well as for prevention of problems during childhood and adolescence. Although universal, the intervention may be useful for parents and adolescents in need of further support and could reduce the use of mental health care resources. The universal aspect of the program could encourage positive family interactions, reducing conflicts in this stage of adolescent independence. If the study implies significant gains, the intervention could easily be offered to parents in general which is beneficial regarding scale-up [49].

The present project promises several new insights and innovations that are noteworthy. Scientific contributions within the area of social emotional learning (SEL) pertain to the six- to eight-year follow up of the PATHS intervention, which will be conducted in this project (i.e., pre-test data). PATHS is a SEL intervention that aims to directly support the development of emotion regulation, a key aspect of social emotional competence. Long term follow ups are rare, especially outside of the U.S., and only two prior studies have followed-up preschool SEL intervention for more than four years. In this project, the long-term impact of an intervention to support preschool children’s social emotional competence will be documented. Furthermore, the project advances our knowledge about developmental trajectories relevant to emotion regulation and mental health, which can inform wider SEL interventions.

Regarding the booster intervention, study of the effects of the Parent Web (PW) contributes to the field of evidence-based parent training interventions, which are generally associated with improved mental health and well-being in children and parents. In previous research, the selected version of the PW intervention showed promising effects, with reductions in parent reported externalizing behavior in youth as well as reduced depressive symptoms and stress in parents [30]. The present trial will investigate whether PW is effective at the universal level. This will lay the groundwork for more evidence-based options for universal parent training with the goal of enhancing mental health and well-being for families with adolescents. Since the PW connects with parents online, it provides an easily accessible parent training program, thus representing an ideal type of intervention. This is especially important considering the COVID-19 pandemic and the subsequent increased demand for greater flexibility via digital services, which may be vital to addressing health inequalities.

Because this is a booster intervention for a particular cohort of families who had prior participation in the preschool edition of PATHS, the generalizability of study findings is not as robust as it would be if this was a one-time new study enrollment with a wider pool of families. Other limitations to the current design are that this is a quasi- rather than randomized controlled trial. Advantages to this study design and trial are that the effects of multiple universal interventions with families are tested, and this trial is tested in an effectiveness trial approach with also gives insight into study recruitment and intervention completion expectations for future intervention trials of PW.

We expect to report project findings in open access scientific publications (e.g., peer reviewed). We also plan to create annual project updates distributed through our project website and to engage in outreach about our study and the topics that are involved in the project (via meetings, a project forum event with community stakeholders). Study results will not be described until all relevant project activities are completed. All results are reported at a group level.

## Supporting information

S1 File Populated SPIRIT checklist. (DOC)

S2 File Translated copy of protocol that was approved by the ethics committee. (PDF)

S3 File Information to participants–Parent Web. (DOCX)

S4 File Participant consent form–Parent Web. (DOCX)

S5 File Participant consent form–control. (DOCX)

S6 File Information to participants–control. (DOCX)
