# Supplementary material for: Study protocol for a non-randomized controlled trial of the effects of internet-based parent training as a booster to the preschool edition of PATHS®: Universal edition of the Parent Web
Source: PLoS One. 2023 Apr 27;18(4):e0284926. doi: 10.1371/journal.pone.0284926 (PMC10138844; doi:10.1371/journal.pone.0284926)
Supplement: S2 File — (PDF) [file pone.0284926.s002.pdf]

Project Title: Children's social and emotional competence and family's well-being when children become teenagers/Barns sociala och känslomässiga kompetens och familjens välmående när barn blir tonåringar

Name of PI: Laura Ferrer-Wreder, Ph.D.,

Host Institution: Stockholm University, Department of Psychology

Etikprövningsmyndigheten
2022-05932-02-325884
2022-11-02

Table of Contents – Research Plan

List of Abbreviations 3

Scientific issues to be investigated 4

Overall purpose, specific objectives, hypotheses and research question 4

General overview and preliminary results 5

Overview related to work package 1 (WP1) – the follow up study of the PATHS cohort children (Group 1) and a matched comparison group of children (Group 2) 5

PATHS: A social emotional learning intervention for children in preschool 5

Overview economic evaluation of the PATHS trial using follow up data for PATHS cohort and data available in the published research literature 6

Preliminary findings from research conducted by our research group: The preschool PATHS intervention trial in Sweden (a completed study) 6

Preliminary findings: Ongoing related study conducted by our research group to develop a new method to understand neighborhood social and economic conditions in urban Swedish contexts 8

Overview related to work package 2 – A booster intervention for PATHS cohort children's parents (Group 1) designed to foster positive changes in parent child relations relative to a matched comparison group (Group 3) 9

Preliminary findings: A study conducted by a member of our research group (Pia Enebrink) to develop the Parent-Web Intervention 10

DESCRIPTION OF PROJECT 11

Project setting 11

Participant Recruitment and Inclusion/Exclusion Criteria 12

Matching Procedure to Identify Group 2 and 3 Participants 14

Changes in addendum Nov. 2, 2022 15

Table 1. Schedule of Assessments/Constructs Measured by Study Arm 17

Assessments Described 20

Child Assessment 20

Overview of the child self-report surveys (completed in person with a research assistant) 20

Overview of the child performance tasks 22

Parent Assessment 23

Parent Assessment for Group 1 parents 23

Parent Assessment for all Parent Groups 1, 2, and 3. 27
Neighborhood assessment and SSO procedure 33
SSO assessment 33
Detailed Procedure for SSO assessment 33
Registry data at the postal code level 34
Universal Parent Education, the Parent Web (Work Package 2) 35
Table 5. Content of the Parent Web (universal edition) 35
Parent Web (PW) Outcomes 36
Sample sizes in the work packages 36
Study designs and intervention allocation 36
Statistical methods 37
Work package 1, main analysis plan 37
Work package 2, main analysis plan 39
Expected outcomes of the project 39
Dissemination of results 40
Ethics and Data Safety 40
Funding and support 43
Key References 43

Etikprövningsmyndigheten
2022-05932-02-325884
2022-11-02

List of Abbreviations

EF = executive functioning

PATHS = Promoting Alternative Thinking Strategies (a social emotional learning curriculum)

RCT = randomized controlled trial

REDI = Research-based, Developmentally Informed (PATHS + literacy training curriculum)

SEC = social emotional competence

SEL = social and emotional learning

SEM = structural equation modeling

SSO = systematic social observation

U.S. = United States

WP = Work package

Etikprövningsmyndigheten
2022-05932-02-325884
2022-11-02

Etikprövningsmyndigheten
2022-05932-02-325884
2022-11-02

Scientific issues to be investigated

Overall purpose, specific objectives, hypotheses and research question

This project has two work packages (i.e., sub-studies) that are inter-related. Each work package has a specific purpose with related hypotheses and/or research questions, as listed below.

Work package 1 (WP1) is a follow up of the PATHS cohort children (Group 1) and a matched comparison group (Group 2). Work Package 1 (i.e., WP1) addresses Purposes 1 and 2, as well as hypotheses 1 through 3, listed below.

PURPOSE 1: Widen the range of available early childhood interventions (i.e., PATHS preschool intervention) that make economic sense, are feasible, and have evidence for long lasting beneficial effects on children’s SEC (social emotional competence) and mental health.

Hypothesis 1: Children participating in PATHS as 4 to 5-year olds will show better social emotional competence (SEC), mental health, and academic performance at age 11 to 13 years old in comparison to a matched sample of children.

Hypothesis 2: The early promotion of children’s social emotional competence (SEC) will be associated with economic benefits. An economic study will be conducted and will use publicly available archival data that already exists in the scientific research literature and newly collected data with the PATHS cohort at age 11 to 13. These newly collected data from group 1 is relevant to both hypothesis 1 and 2.

PURPOSE 2: To identify the ways in which the socio-economic conditions of the neighborhoods that children live in are important to children's social emotional competence and mental health.

Hypothesis 3: Across cohorts (in the original PATHS cohort and matched sample, Groups 1 and 2), elevated social emotional competence (SEC) in children will buffer the effects of contextual risks at the neighborhood level, and will be associated with better mental health.

Work package 2 (WP2) is a booster intervention (i.e., an intervention trial) for the PATHS cohort children’s parents (Group 1). The effects of the Parent Web booster intervention will be determined relative to a matched comparison group (Group 3). Work Package 2 addresses Purpose 3, as well as hypothesis 4 and research questions 1 and 2, which are listed below.

PURPOSE 3: To develop a beneficial and accessible online intervention for parents of adolescents. To achieve this aim, this project will implement and test the effects a Swedish developed parent training intervention called the Parent Web (Wetterborg, Enebrink, et al., 2019). The universal edition of the Parent Web will be tested with a quasi-experimental design with PATHS cohort children’s parents (Group 1) as the immediate intervention group relative to a comparison group of parents (Group 3) who are in a wait-list control condition.

Hypothesis 4: Relative to parents in a matched wait list comparison condition (Group 3), parents taking part in the Parent Web (PATHS cohort children’s parents, Group 1) will show significant pre to posttest benefits on emotional well-being, improved parenting strategies, perceived warmth, as well as reduced family conflicts, parental stress, and parent reported youth externalizing and internalizing problems.

Research Question 1: Do subgroups of parents/families (e.g., parents whose children are girls or boys) differentially benefit from the Parent Web? A prior intervention trial of PW indicated families with adolescent girls, relative to adolescent boys, particularly benefited from the intervention (Wetterborg, Enebrink, et al. 2019).

Research Question 2: How do parents view the Parent Web in terms of its acceptability?

General overview and preliminary results

Overview related to work package 1 (WP1) – the follow up study of the PATHS cohort children (Group 1) and a matched comparison group of children (Group 2)

Work package 1 (WP1) is a follow up study that involves the PATHS cohort children (Group 1) and a matched comparison group (Group 2). The fundamental subject matter of WP1 is children's social emotional competence (SEC).

SEC in childhood are psychological assets essential to children's ability to thrive internally, as well as help children navigate complex and developmentally important social relationships, with peers and adults. This project's theory of SEC is CASEL'S five competence domain model (Weissberg et al., 2015). The CASEL model views SEC as multidimensional and made up of positively related (i.e., overlapping) yet in some regards still distinct domains namely: Self-awareness, self-management, relationship skills, social awareness, and responsible decision making (Weissberg et al., 2015).

This project at the widest conceptual level is guided by theory and research on the positive development of young people (e.g., Eichas, Ferrer-Wreder, & Olsson, 2019; Tolan et al., 2016). In this project, we aim to identify new ways to promote psychological strengths in young people and/or to increase contextual resources (at home, within families) that can foster the overall development and well-being of young people, and to protect against social adjustment problems and mental ill health.

Etikprövningsmyndigheten
2022-05932-02-325884
2022-11-02

PATHS: A social emotional learning intervention for children in preschool

Promoting Alternative THinking Strategies (PATHS) is an evidence-based social emotional learning (SEL) intervention originally developed in the United States (U.S.; Domitrovich, Cortes, & Greenberg, 2007). The preschool edition of PATHS is a teacher implemented universal intervention, in other words the intervention is designed to be suitable for all children attending preschool and the intervention is not focused on particular subgroups of children who may experience more adversity (Domitrovich et al., 2007).

Preschool PATHS is delivered as a teaching curriculum plus in-person training and a coaching/support model for teachers (Fishbein et al., 2016). PATHS is based on theories (e.g., emotional intelligence) and research on SEC as well as knowledge about brain development, and theories about how school environments can support children's SEC (Domitrovich et al., 2007). PATHS consists of 33 lessons that use children's literature, puppets, role plays, and activities that help children learn about emotions within and outside of time formally dedicated to PATHS, and PATHS lessons are given once a week over the course of a school year (Domitrovich et al., 2007; Fishbein et al., 2016).

Preschool PATHS has been tested in the U.S. with rigorous research designs (e.g., randomized controlled trials, RCTs; Domitrovich et al., 2007). In one RCT, PATHS was paired with literacy training. This intervention and its trial are called REDI (Research-based, Developmentally Informed;

Bierman et al., 2008a, 2008b; N=356). In REDI, immediate intervention benefits were found for vocabulary, literacy skills, emotional knowledge, and improved social cognitions (Bierman et al., 2008b). Five years after the intervention, analyses showed long-term intervention benefits on academic outcomes. Children who began behind their peers in executive functioning (EF) skills at the beginning of the study particularly benefited from the intervention (Sasser et al., 2017). In REDI, a booster intervention was also conducted and this intervention focused on PATHS children's parents and how they can support their child's SEC and literacy.

In our new project, we derive our inspiration to conduct a follow up study plus a booster intervention from the REDI trial (Bierman et al., 2008a). However, we tailored the booster intervention in this project to be best suited to early adolescence (families parenting 11 to 13-year olds), because early adolescence is the developmental period that our study participants will be in, when this project is conducted. And in our project's booster intervention, the focus of intervention moves to parents as a support for children as they make the transition into adolescence with some of the key developmental tasks of adolescence in mind (e.g., realignment of parent-child relationship in adolescence, managing other complex social relationships inside and outside families, balancing autonomy and responsibility in the parent-adolescent relationship). This overview provides a theoretical and empirical basis Purpose 1, hypothesis 1 in work package 1.

Overview economic evaluation of the PATHS trial using follow up data for PATHS cohort and data available in the published research literature

Etikprövningsmyndigheten
2022-05932-02-325884
2022-11-02

Prior research on the economic impact of interventions aiming to enhance children's SEC is limited in terms of the number of studies available (Belfield et al., 2015). A recent large-scale meta-analysis of school-based programs to promote child and adolescent SEC (Taylor, Oberle, Durlak, & Weissberg, 2017) identified 82 school-based, universal ***social and emotional learning (SEL)*** programs that had an at least a six month follow up assessment. The 82 interventions involved 97,406 children and adolescents. Investigators found that youth who took part in SEL interventions, relative to those in control groups, demonstrated significantly improved social-emotional skills, attitudes, and well-being. Of the SEL interventions included in this meta-analysis, only six had reported information in regards to economic impact, and life time monetary benefits or cost savings per event avoided ranged from 9,940 to 3.9 million U.S. dollars (2015 dollars). SEL interventions have economic benefits (Belfield et al., 2015; Taylor et al., 2017), however there remain few rigorous economic evaluations of interventions designed to impact SEC, and do to this lack of attention such interventions are likely to be overlooked as a key public health strategy (Olsson et al., 2014). This is partly due to the difficulty in understanding and documenting the value of SEC (Belfield et al., 2015). SEC is considered a valuable intervention outcome in its own right. Yet, there is also compelling international longitudinal evidence in favor of the supposition that SEC has an explanatory role in a diversity of public health outcomes, including mental illness. The economic analysis to be conducted as part of Work Package 1 (namely Purpose 1, hypothesis 2) addresses this research and societal priority.

Preliminary findings from research conducted by our research group: The preschool PATHS intervention trial in Sweden (a completed study)

This new project builds off the preschool PATHS intervention trial that was conducted in Sweden from 2012 to 2016. The trial was funded by the 1st call on Children's Mental Health funded by VR, FORTE, et al. (dnr: 259-2012-71) and received ethical approval by the Stockholm Regional Ethical Board on 2012-11-08 (dnr: 2012/1741-31/5). The trial registration number for the preschool PATHS intervention trial is located at the website: ClinicalTrials.gov is NCT04512157.

Etikprövningsmyndigheten
2022-05932-02-325884
2022-11-02

This completed intervention trial of PATHS took place in preschools in municipalities located in region Stockholm. See Eninger et al. (2021) for a detailed description of the intervention trial and study results. The participating municipalities were selected to reflect contrasting socioeconomic conditions at the neighborhood level. The intervention trial was a two-wave pre to posttest, randomized controlled trial with multi-method and informant assessment. Assessments included teacher reported surveys, child tasks, observations of children at play. Participants were 290 four and five-year-old children; 145 wait-list control and 145 intervention) attending 26 preschools (12 intervention, 13 control, 1 school served as control in wave 1 and intervention in wave 2). Within the three municipalities, recruited preschools were cluster randomized into an immediate intervention or a wait-list control group. The unit of assignment and analysis was at the school building level. Intervention dosage and adaptation were measured by teacher report. Intervention fidelity was measured by teachers and observers.

The objectives of the trial were to: (1) conduct a cultural adaptation of PATHS to a Swedish preschool context. For a detailed description of the cultural adaption process see Ferrer-Wreder et al. (in press). The other objective of the trial was to: (2) evaluate the immediate pre to posttest effects of PATHS (Eninger et al. 2021). The most central, primary outcomes targeted for change were improvements in SEC and it was hypothesized that children attending schools in which PATHS was being taught, relative to children in a wait-list control schools, would show pre to posttest improvements in SEC, measured through different assessments.

In the beginning years of the project, the cultural adaptation of PATHS was completed, and attention then turned to testing the immediate effects of PATHS in relation to changes in children's SEC and social adjustment. The outcome results are reported in detail in Eninger et al. (2021). Here, we provide an overview of the main findings from the intervention trial.

Returning back to one of the main theoretical models that guides our work in general, the CASEL model of SEC (Weissberg et al., 2015), outcome analyses showed immediate (pre to posttest) intervention-related changes in four out of five CASEL SEC domains. One domain self-awareness involves, in part, identifying emotions and results showed that PATHS children's emotional knowledge improved relative to children in the control condition (effect sizes across analyses ranged from .22 to .23). Moderation analysis by gender showed that girls in PATHS schools, relative to girls in control schools, particularly benefitted in terms of gains in emotional knowledge (effect size was .55, which is a substantial effect size). Further, girls in PATHS schools, relative to girls in control schools, showed reduced anxiety/somatic symptoms as rated by teachers (effect size was -.35). There were no other results that indicated significant moderation of intervention effects by children's gender (Eninger et al., 2021).

The intervention trial also showed intervention benefits, this time in the CASEL domain of self-management. Here, intervention benefits were found on an index of executive functioning, namely working memory. Children in PATHS schools showed improved working memory, relative to children in the control schools (effect sizes ranged from .36 to .39). In different CASEL domains, specifically, social awareness and relationship skills intervention benefits were found on observer-rated prosocial skills across two play occasions. This is a particularly robust finding given the assessment that is involved, specifically the play observation consists of ratings of children's prosocial skills conducted by two research assistants observing children at play with other children (3 children in total) on two standardized play occasions (Eninger et al., 2021).

Other intervention trial results showed an unexpected, hypothesis inconsistent intervention related increase in teacher reported hyperactivity/impulsivity (effect sizes ranged from -.30 to -.25). This type of effect for hyperactivity/impulsivity has not been shown in any other published preschool PATHS trials to date. Unadjusted means for PATHS children went from .69 at pretest to .86 at posttest, relative to control children means which were .63 at pretest and .55 at posttest. This indicates average

scores for all children in general close to the response option 1 which was "*Sometimes"* on a four-point scale (response options are 0 = *Never/Rarely*, 1 = *Sometimes*, 2 = *Often*, and 3 = *Very Often*). Thus, the level of hyperactivity/impulsivity is overall at the lower end of this continuum. This result could be a potential short-term adverse effect of the intervention, even though the positive benefits associated with PATHS in this intervention trial far outweighed this single potential adverse effect. This finding in regards to hyperactivity/impulsivity will be included in our new project and we plan to examine hyperactivity/impulsivity as part of the follow up study, so that any long effects among PATHS children in this social adjustment domain are well understood and documented.

Our research group's overall interpretation of the meaning of the original Swedish PATHS trial results is that given the array and weight of the positive intervention-related benefits, this unexpected finding on teacher-rated hyperactivity/impulsivity, while important is outweighed by intervention benefits in key aspects of SEC, namely increased emotional knowledge, working memory, prosocial skills, and unique intervention benefits for girls in emotional knowledge and anxiety/somatic symptoms. Increased risk for poor mental health among Swedish adolescent girls is a public health concern and setting children on a strong path into adolescence is of vital importance. Thus, the gender-moderated effect for anxiety/somatic symptoms (an indicator of internalizing problems), showing larger intervention-related decreases for PATHS girls relative to girls in the control group, is particularly important finding, that requires follow up in the present project as well. The PATHS Swedish intervention trial is a completed study and PATHS will not be not implemented as part of the current project for which we are seeking ethical approval (i.e., WP1, WP2). However, our new project builds directly off of this completed intervention trial and one main aspect of our new project is to follow up with the children who took part in the immediate PATHS intervention condition (e.g., WP1 with PATHS children and their parents).

Etikprövningsmyndigheten
2022-05932-02-325884
2022-11-02

Preliminary findings: Ongoing related study conducted by our research group to develop a new method to understand neighborhood social and economic conditions in urban Swedish contexts

As part of a doctoral project conducted by Ingela Clausén Gull (ethical approval, dnr 2018/2334-31/5) at Stockholm University in the Department of Psychology, we continue to examine the intervention effects that took place in the original intervention trial for preschool PATHS. We are in the process of conducting analyses that examine moderation of intervention effects by socioeconomic status of the immediate neighborhoods surrounding the preschools that were involved in the Swedish PATHS trial. We are triangulating registry data regarding local inhabitants' education and income at the postal code level with in person and virtual (through the use of Google Street view) systematic social observation (SSO) of neighborhood characteristics (i.e., streets and green spaces).

The SSO methods that will be used in our new study, are being pilot tested in this ongoing and separate doctoral project that makes use of the already collected data from the Swedish PATHS intervention trial. The connection between this doctoral project and our new project is that the SSO methods developed in this doctoral project will be used to measure neighborhood level characteristics of importance to youth development in terms of participating children's SEC and mental health, which relates to Purpose 2, hypothesis 3. Thus, the aim is to examine if elevated levels of children's SEC can function as a protective factor in the context of increased neighborhood level adversity, and thereby be associated with better mental health among children in Groups 1 and 2 (PATHS cohort children and a matched comparison group of children).

Work in the PATHS trial in Sweden involved examination of preschool climate and neighborhood context. In prior research conducted outside of Sweden, contextual factors found in neighborhoods have been associated with problem behaviors as well as child development and other educational and health outcomes (e.g., Odgers et al. 2012). Consistent with our own research focus, much of this

research has considered contextual factors as likely important moderators of the effectiveness of prevention efforts.

Neighborhood strain can, for example, have direct and indirect effects on youth in relation to prevention efforts but also, as children age and become more independent, interactions with neighbors increase and social stigmatization or social capital attributed to one's immediate neighborhood can take on increased importance. Current Swedish research on registry derived indicators of neighborhood factors have been connected to income inequality, school choice and segregation, physical health, crime and immigration patterns, yet little direct assessment of neighborhood factors (e.g., risks/strengths in the built environment and in resident perceptions of their neighborhood) have been made in relation to understanding individual level child development and mental health. This gap in the research literature is addressed by work package 1, purpose 2, hypothesis 3. Understanding neighborhood risk/protection and their connections to child SEC and how such competencies may buffer risk in a Swedish context is valuable in a number of respects including informing the design of future coordinated multi-level (i.e., person-context) interventions.

The SSO methods that we will use are derived from several prior studies, for example a longitudinal study of children in the United Kingdom (UK) was a main model for our project's use of SSO measurement methods. This UK study was called the E-Risk Longitudinal Twin study (Odgers et al. 2012). Odgers et al. (2012) used a standardized virtual SSO protocol with multiple raters and found that decay and deterioration (rated physical disorder such as graffiti, vandalized signs, deteriorated apartment buildings, and a lack of green spaces, perceived danger) in the neighborhood in the immediate vicinity of participating children's homes was significantly related to antisocial behavior in study children at age 12 and the method significantly and positively correlated as expected with socioeconomic status and a resident survey of social cohesion. The research studies ongoing by Ingela Clausén Gull for her dissertation in the context of the original Swedish PATHS trial has allowed us to develop a standardized SSO virtual protocol that has good reliability and been pilot tested and is well suited to the Swedish residential contexts relevant to present study.

Etikprövningsmyndigheten
2022-05932-02-325884
2022-11-02

Overview related to work package 2 – A booster intervention for PATHS cohort children's parents (Group 1) designed to foster positive changes in parent child relations relative to a matched comparison group (Group 3)

Work package 2 (WP2) is a booster intervention/intervention outcome trial. WP2 participants are the PATHS cohort children's parents (Group 1) who will be in an immediate intervention group taking part in the Parent Web (PW) intervention trial. The effects of PW will be determined relative to a matched comparison group (Group 3). Only parents are the participants in WP2.

The Parent Web is part of the larger research field on parent training interventions. Intervention science is advancing. New interventions are being developed that boost individual and relational psychological strengths within adolescents and their families. Internal psychological assets/strengths within the adolescent include social emotional competence and contextual assets/strengths include ***connection*** to important others including parents and caregivers (Tolan et al., 2016).

Decades ago, interventions were narrowly designed to reduce a single problem behavior and this approach left institutions such as schools with a heavy burden to implement resource intensive, uncoordinated interventions (Botvin & Griffin 2014). Further, empirical evidence from longitudinal studies shows a complex relationship between social problems, mental ill-health, developmental assets, and well-being. Interventions of today (and the future) are built to capitalize on this complex relationship and aim to work holistically in a more general, universal way to boost the chances of success and health for large numbers of youth and their families. While it is clear that targeted interventions for some groups of high-risk youth will always be needed, interventions are also needed

that are broad, wide reaching in scope and aim to boost psychological and relational assets essential to youth development and well-being. The new generation of interventions should also be culturally competent and easy to access/use. The thinking behind today's youth development and prevention programs is that by building up broad yet fundamental assets, one is raising the chances that young people and their families will competently navigate and even thrive during the expected and unexpected challenges of life (Tolan et al., 2016). Our broader program of research, in which the trial of the Parent Web is embedded, aims to lead in the development of next generation interventions. The Parent Web (and PATHS) fits many of the qualities outlined above as *Next Generation*.

Within the wider field of parent training interventions internationally as well as in the Stockholm region, there are many evidence-based parent training programs (Florean et al., 2020). This is because the family plays a major role in children's and adolescents' mental health and well-being (e.g., Parke, 2004). However, most of the parent training programs that are currently available that have shown positive benefits with youth who experience elevated risk (e.g., Family Check Up, Komet) and/or for young children (e.g., Alla Barn i Centrum, universal for children). For example, Alla Barn i Centrum is a universal health-promoting program developed in Sweden for parents with children aged 3-12 years. This parent training intervention has four meetings and a booster meeting and has been shown to have an effect on children's physical and mental health (Ulfsdotter et al., 2014), up to 6 months after the end of the intervention.

Unfortunately, for parents with children who are in their adolescent years, there are generally fewer evidence-based beneficial parent training interventions, compared to interventions designed for parents with young children. Internationally, there are programs such as Triple P for teenagers (Chu et al, 2014), Parents Plus Adolescent Program (PPAP, Nitsch et al, 2015), ABCD parenting young adolescents (Burke et al, 2012). Thus, there presently exist few evidence-based parent training interventions that are universal for a general population of parents and *adolescent children,* are geared towards the promotion of positive youth development and universal prevention, are available online, and are culturally tailored for a Swedish parenting context. The Parent Web fits all of these criteria and fills this specific research and societal need.

Etikprövningsmyndigheten
2022-05932-02-325884
2022-11-02

In general, why are parent training interventions of benefit? The relationship between parents and young people is an important factor that supports the overall development and mental health of family members (Parke, 2004). A universal parent training program that is given widely to all parents can be one of several ways to help parents to be a positive support for their children and promote good development. Universal parent training/education can be an opportunity to reach parents who want help as their child transitions into adolescence and meets the challenges related to a realignment of the parent-child relationship (e.g., navigating some normative increases in risk taking behaviors, new romantic relationships, and new autonomy and responsibilities for youth during adolescence that were not as pressing in childhood). Universal parent training interventions also promise to be an aid to parents who are also experiencing challenges related to general circumstances such as changes in work, finances, or partner relationships, and health.

Preliminary findings: A study conducted by a member of our research group (Pia Enebrink) to develop the Parent-Web Intervention

In Sweden, the Parent Web (PW; Lönn-Rhodin, Forster, & Enebrink, 2015) was developed a few years ago, as an online web-based prevention-parental support program for parents with teenagers (11 to 17-year olds) in which there are many conflicts (i.e., as a selected intervention). Its aim is to support parenting practices and parent-adolescent interactions that encourage prosocial behavior and emotional regulation in youth, reduce instances of coercive parenting that encourage aversive, negative escalatory interactions in families, improve assertive communication and problem solving in families as well as increase warmth and closeness among parents and adolescents (Wetterborg,

Enebrink, et al. 2019). PW is now available in universal and selective versions, both of which are derived from social learning and coercion theory.

Presently, there are no published outcome evaluations of the universal PW. However, the effects of the selective edition of PW have been tested in a randomized controlled trial with 43 parents in an immediate intervention condition and 32 parents in a wait-list control condition. The assessment in the trial had pre, post, and follow up testing after 6 to 9 months; Wetterborg, Enebrink, et al. 2019). In this intervention trial, intervention (relative to control) parents reported reductions in externalizing problems in youth, less depression and stress in parents, more family warmth, and less family conflict. Moderation analyses indicated that families with adolescent girls, relative to adolescent boys, particularly benefited from the intervention (Wetterborg, Enebrink, et al. 2019). This overview provides a theoretical and empirical basis Purpose 3, hypothesis 4, research questions 1 and 2 in work package 2.

DESCRIPTION OF PROJECT

Project setting

This study is managed and based at Stockholm University (SU) in the Department of Psychology. Online data collection for the parent assessment and the implementation of the PW are based at Karolinska Institute, Clinical Neuroscience, Division of Psychology in collaboration with Dr. Pia Enebrink. The departments involved have ample facilities to conduct research and long-standing research and outreach ties to Region Stockholm as well as other stakeholders in the local community. Our team is well experienced and interdisciplinary, with researchers representing the disciplines of psychology, social work, public health, and special education.

Etikprövningsmyndigheten
2022-05932-02-325884
2022-11-02

The key project team members are consistently involved in overall project supervision. Dr. Laura Ferrer-Wreder (project leader) has extensive research experience conducting intervention trials and longitudinal studies, as well as has recently led and completed a home-based study with families who have adolescents (Ferrer-Wreder et al., 2020b, Stockholm regional ethics approval, Dnr. 2016/483-31/5). The families in this descriptive pilot study were identified and recruited through registry data and in collaboration with Statistics Sweden (Ferrer-Wreder et al. 2020b), which is relevant project experience that supports the use of registry-based research procedures to be used in this new project.

Other members of the research team have extensive experience with intervention trials and youth development (Drs. Tina Olsson, Göteborg University; Lilianne Eninger, SU; Åsa Norman, SU/KI, Hanna Ginner Hau, Licensed psychologist) and other team members also have special competence in parent-child relations, mental health, as well as online and in person interventions for parents (Dr. Pia Enebrink, Licensed psychologist, Karolinska Institute; Dr. Lene Lindberg, Licensed psychologist, Karolinska Institutet & Center for Epidemiology and Community Medicine Region Stockholm; Dr. Sabina Kapetanovic, SU/University West; Dr. Johanna Stålnacke, Licensed psychologist, SU/Göteborg University).

Etikprövningsmyndigheten
2022-05932-02-325884
2022-11-02

Participant Recruitment and Inclusion/Exclusion Criteria

In this project, there are three groups of participants.

Group 1. As 4 to 5-year olds, Group 1 (n = 144[1]) were part of the immediate intervention group in the Swedish preschool PATHS trial. As noted, the original trial tested the pre to posttest effects of PATHS with a primary focus on strengthening children's social emotional competence (SEC). The current project has sub-studies (Work Package 1) that will provide a longitudinal follow up of the PATHS cohort children (Group 1) to see how they are faring several years after the original intervention in preschool.

Study inclusion criteria for Group 1:

- *The child took part in the Swedish PATHS intervention trial, in the immediate intervention group, and their parents consented to have future contact with the PATHS research group.* This is the case for 144 children.

- *The child and participating parents presently live in Sweden.*

On the parental informed consent form for the Swedish PATHS trial (this is a completed study), parents could indicate if they wished to have future contact with our research group regarding PATHS. One family indicated that they did not wish to have future contact, 19 parents left this alternative blank (no response indicated to this question), and 125 parents indicated "yes" that they would like to have future contact. We will update the information regarding parents' and child's name, home address, and/or phone numbers to parents that we presently have from the original Swedish PATHS trial. This will be done except for the one family that did not want future contact (n = 144 children and their parents/caregivers/legal guardians). This information was provided by parents on the original intervention trial's parental consent forms. This information is archival data that exists for all PATHS families.

We will update the PATHS family contact information with the following procedure:

a) first attempt to update the contact information for families will be through existing public websites (e.g., eniro; hitta), if this does not provide accurate information, then

b) we will request updated contact information for participating families based on registry information from Statistics Sweden

c) final attempt to update contact information will be made is through contacting families with the original contact information they provided during the original intervention trial

When directly contacting families, the first contact will be by written letter to home addresses via regular post, subsequent contacts in the case of non-response will be by phone, or text by phone number.

[1] The published outcome study of the original PATHS project (Eninger et al., 2021) 140 children were included in the immediate intervention condition outcome analysis, five children were recruited into the study but were not included in this outcome analysis due to complete missing data on, one or more assessments, we plan to include the complete cohort of children in this new study, if in the original consent form parents indicate that it was okay for the research team to contact them in the future, this is the case for 144 children from the original PATHS immediate intervention cohort.

In our research team’s communication with Group 1 parents we will:

We will provide an update to parents about the results of the original preschool PATHS trial, via letter and/or verbally. After explaining the original study trial results and answering any questions or reflections of the participants (parents and/or children), we will then explain the new project and ask for parents’ and the participating child’s consent to take part in the new project.

If families consent to participate in the new study, we will ask parents to update contact information for the family: Namely parents’ and child’s name, home addresses, and phone numbers of parents/current legal guardians. This information is recorded on the consent signature form for Group 1 parents. This is the recruitment procedure for Group 1.

Group 2. In order to examine the possible long-term effects of preschool PATHS, a matched comparison group of children and their parents will be recruited into this project as Group 2. Groups 1 and 2 participate in the follow up study (i.e., Work Package 1).

Inclusion criteria for Group 2

- *Child participants in Group 1 and 2 are matched on the matching criteria (see matching criteria procedure). Group 1 and 2 parents will not be matched, but will only be required to be parents/legal guardians of the matched children. We will ask that only one parent per family take part as a participant in Group 2, if there are two parents, parents choose which parent takes part in this project. There are no other inclusion/exclusion criteria for Group 2.*

Etikprövningsmyndigheten
2022-05932-02-325884
2022-11-02

Group 3. Another matched comparison group will be recruited as Group 3. Children in Group 1 and Group 3 are matched on the matching criteria (see matching criteria procedure). Only parents will be recruited as study participants into Group 3. Parents in Group 1 and Group 3 take part in the intervention trial of the Parent Web (i.e., Work Package 2). Group 3 parents are recruited as participants in a wait list comparison condition (non-random assignment to condition) in the Parent Web Intervention trial. Group 1 parents after completing the follow up assessment will take part in the Parent Web (WP2), and this will be in an immediate intervention group (Group 1 parents) for the Parent Web, which provides a developmentally well-timed booster intervention for PATHS families, and also tests the effects of this universal parent training intervention. If Group 1 parents consented to participate in WP1 and WP2, they will enter into WP2 (the Parent Web trial) on a rolling basis as they finish the assessment for WP1.

Inclusion criteria for Group 3

- *Children in Group 1 and Group 3 are matched on the matching criteria. Group 1 and 3 parents will not be matched. But will only be required to be parents of the matched children.*

- *All matched parents in Group 3 can participate in work package 2 (the Parent Web intervention trial). There are no other inclusion/exclusion criteria for this group.*

Although the children in Group 3 are matched to Group 1 children, only adults (parents of the Group 1 and 3 children) take part in the intervention trial of the Parent Web (i.e., Work Package 2).

Matching Procedure to Identify Group 2 and 3 Participants

A recruitment pool of families will be identified with the assistance of Statistics Sweden. Examples of registries that can be used for the matching procedure by Statistics Sweden include the Total Population Register (Registret över totalbefolkningen, RTB) and the Swedish Multi-generation Register (Flergenerationsregistret). Statistics Sweden will identify a matched recruitment pool of families and provide contact information for the matched families to the research team (parents' and matched child's name, phone number, matched child's home address). The research team is responsible for recruitment of all study participants. Statistics Sweden has provided consultation to develop this matching procedure.

The participant pool for Groups 2 and 3 will allow for the identification and then recruitment of the participants in Groups 2 and 3. The matching criteria to create the participant pool are:

1. Group 2 and 3 child's present-day gender is the same as the matched child in Group 1.
2. Group 2 and 3 child's present-day age is similar to the matched child in Group 1 with child birthdays within 6 months of each other.
3. Group 2 and 3 children live in the same postal code as the matched Group 1 child.
4. Matched children have all lived in Sweden since 2014.

All four matching criteria need to be fulfilled in order to be in the matched recruitment pool.

If these 4-points for the matching criteria are too restrictive and do not provide enough matches to recruit from, then the matching criteria will be reduced to points 1, 2, and point 3.

We will ask Statistics Sweden to identify all possible matches, first using the 4-point matching criteria. Then, we will examine the number of all possible matches for all children in the PATHS cohort. Our decision rule for keeping the original matching criteria (4-points listed above) would be that at least 8 matched children are identified per PATHS child, this anticipates a minimum 25% participation rate for families in Groups 2 and 3, if two matched families are recruited out a potential total of 8 matched children identified through the matching criteria.

Recruitment of participants in Groups 1, 2, and 3 will occur during the same two-month time period. If there is a drop out of a matched family in Groups 2 or 3 during the course of the project period, a new matched family will be recruited to replace the match that has dropped out.

Etikprövningsmyndigheten
2022-05932-02-325884
2022-11-02

Figure 1. Study Arms/Groups Described (figure is removed)

Figures 2a and 2b Participant Timeline and Measurement (figures are removed)

Figures 1, 2a, and 2b replaced with text below submitted as an ethics addendum application in November 2, 2022

Change 1

Group 1 is the cohort of children who took part in the original Swedish PATHS intervention trial and were an immediate intervention group of 144 children. In this project, group 1 children and one of their parents are in the first study arm. Group 1 children recruited into the current project are up to 144 and one of their parents (n = 144 parents). There is no change in the proposed number of participants for this study arm.

Group 2 is the second study arm for this project. Group 2 is a matched comparison group that participates in the follow up study (and does not participate in the Parent Web). We propose to be able to have two families (instead of one family) per Group 1 family (i.e., PATHS family). Group 2 children will be up to 288 and one of their parents (n = 288 parents).

Group 3 is the third study for this project. Group 3 parents will take part in the Parent Web intervention trial. We propose to be able to have two families (instead of one family) per Group 1 family. Group 3 parents will be up to 576 parents. In this study arm, was originally proposed that one or both parents in a matched family can participate in the Parent Web. Parents decide if one or two parents per family participate. Thus, this 576, counts for a maximum number of participants in Group 3 if both parents in the two matched families decide to participate in the Parent Web.

Etikprövningsmyndigheten
2022-05932-02-325884
2022-11-02

Change 2

Child assessment/data collection with both child survey and cognitive tasks will take place primarily online. Administration of the child assessment requires a trained adult/researcher to be present so that children understand the survey and cognitive task before they perform it so that children can ask questions about how the survey or cognitive task works, if they have such questions. We have piloted the child assessment and have developed an online protocol in which a researcher can be present online (on zoom, web-based online meeting platform supported by Stockholm University) when the child completes the survey and cognitive tasks.

The online assessment with child participants in Groups 1 and 2 will take place on a mutually agreed upon day, time and location (i.e., agreed upon by parents, the child, and research team) where the child can be relatively undisturbed during the assessment occasion. Locations for the child assessment can include the child's home with one or more parents present when the assessment occurs. Participating children will use a secure cloud (the same cloud described for the parent surveys will be used for the child surveys) and the cognitive task data will be in a cloud that is developed by the task developers. Data from the cognitive tasks will be downloaded on a daily basis into a secure encrypted database located at Stockholm University at the Department of Psychology that is managed by the research team.

The reasons for changes

Change 1:

This additional number of matched families will allow for a more robust and well powered (statistically) analysis of the original hypotheses and research questions in this study.

Change 2:

An unexpectedly large number of participating families live outside of Stockholm area, including municipalities such as Enköping and Lund. This particularly is the case for families in group 1, the original cohort of Paths families. In addition, during recruitment of group 1 families, a few families have expressed a wish not to be assessed in school. Some of the families have expressed a wish to be assessed at home. Instead of assessing the children differently (some in school, some at home, some online) and in that sense risking both the increased attrition and measurement error, we believe that a coherent online child assessment would be more feasible and less time and resource consuming.

Etikprövningsmyndigheten
2022-05932-02-325884
2022-11-02

Table 1. Schedule of Assessments/Constructs Measured by Study Arm

**GROUP 1: Children and Parents**			
	**Data collection**		
**Type of Information Collected**	**Group 1, Time 1 (G1, T1) Fall 2022**	**During Parent Web Intervention**	**Group 1, Time 2 (G1, T2) Winter, Spring, Summer 2023**
**Child assessment (in person data collection computerized)**			
Y1. SSIS-SEL	X		
Y2. SDQ	X		
Y3. SAQ	X		
Y4. C19	X		
Y5. Stroop task	X		
Y6. Hearts and Flowers task	X		
Y7. Attention task	X		
**Parent assessment (online)**			
P1. PD	X		
P2. SSIS-SEL	X		
P3. SDQ	X		X
P4. DBD	X		X
P5. FCU	X		X
P6. PARCA	X		X
P7. PSS	X		X
P8. HAD	X		X
P9. ASQ	X		X
P10. C19	X		
P11. Imp		X	
**Neighborhood assessment**			
N1. SSO, online/in person	X		
N2. Residential income and highest education of inhabitants at the postal code level, registry data	X		

Notes. **Child assessment:** Y3. SAQ. School adjustment questionnaire (CPPRG, 1997); Y5. Stroop task (Duell et al., 2018); Y6. Hearts and Flowers Task (Watts et al., 2018). ***Scales with parallel editions for children and parents***: Y1/P2: SSIS-SEL Brief survey, Student and Parent forms (Anthony et al., 2020a; Elliott et al., 2021); Y2/P3: SDQ. Strengths and Difficulties Questionnaire, Youth and Parent forms (Goodman, 1997); Y4/P10: Experiences Related to COVID-19 instrument (Skinner & Lansford, 2020). **Parent assessment:** P1. *PD*, Parent Background Information (Wetterborg et al. 2019), P4. *DBD*, Oppositional Defiant Scale of the Disruptive Disorder Rating Scale (Pelham, et al., 1992); P5. *FCU*. FCU Caregiver Assessment Scale (Criss, & Shaw 2005); *P6. PARCA*, *Parenting Children and Adolescents Scale* (Ringle et al., 2019); *P7. PSS*, The Perceived Stress Scale (Cohen et al., 1983); *P8. HAD*, Hospital Anxiety and Depression Scale (Zigmond & Snaith, 1983). *P9. ASQ*, Affective Style Questionnaire (Hofmann & Kashdan, 2010). SSO assessment. ***N1. SSO***, Systematic social observation (SSO) of neighborhood features that specifically are 37 observer rated items measuring physical disorder, availability of recreation, type of residences, condition of signs and communication, availability of businesses and services, overall condition of a specific geographic area (Odgers et al., 2009, 2012; Quinn et al., 2016).

Etikprövningsmyndigheten
2022-05932-02-325884
2022-11-02

Table 1. Schedule of Assessments/Constructs Measured by Study Arm (continued)

GROUP 2: Children and Parents	
	Data collection
Type of Information Collected	Group 2, Time 1 (G2, T1) Fall 2022
**Child assessment (in person data collection computerized)**	
Y1. SSIS-Brief, Student	X
Y2. SDQ, Youth	X
Y3. SAQ	X
Y4. C19, Youth	X
Y5. Stroop task	X
Y6. Hearts and Flowers task	X
Y7. Attention task	X
**Parent assessment (online)**	
P1. PD	X
P2. SSIS-SEL	X
P3. SDQ, Parent	X
P10. C19P, Parent	X
**Neighborhood assessment**	
N1. SSO assessment, observer rated online and in person	X
N2. Residential income and highest education of inhabitants at the postal code level, registry data	X

Etikprövningsmyndigheten
2022-05932-02-325884
2022-11-02

Table 1. Schedule of Assessments/Constructs Measured by Study Arm (continued)

GROUP 3: Parents				
	Data collection			
**Type of Information Collected**	**Group 3, Time 1 (G3, T1) Fall 2022**	**Group 3, Time 2 (G3, T2) Winter, Spring, Summer 2023**	**During Parent Web Intervention**	**Group 3, Time 3 (G3, T3) Fall 2023**
**Parent assessment (online)**				
P1. PD	X			
P3. SDQ, Parent	X	X		X
P4. DBD	X	X		X
P5. FCU	X	X		X
P6. PARCA	X	X		X
P7. PSS	X	X		X
P8. HAD	X	X		X
P9. ASQ	X	X		X
P10. C19P, Parent	X			
P11. Implementation/consumer satisfaction survey			X	

Etikprövningsmyndigheten
2022-05932-02-325884
2022-11-02

Assessments Described

All assessments used in this project are well-validated in prior research studies. If the instrument was originally in English it was translated into Swedish through forward and back translation by qualified researchers on our research team. All assessments will be further piloted tested before used in this project, and refined based on the pilot testing.

Child Assessment

Work Package 1– Child Measures/Assessment

The child measures will be used only in the follow up study (i.e., work package 1, for Groups 1 and 2 children). Children will complete a computerized or paper and pencil battery of standard surveys measuring main study constructs, namely child's social emotional competence (SEC) and mental health/well-being. In addition to the surveys, children will also be asked to complete tasks that measure executive functioning, which includes working memory, inhibition, cognitive flexibility, and attention. Executive functioning is an important indicator of social emotional competence.

For this project, the child assessment will be a one-time data collection that is expected to take place in Fall 2022. Demographic information for all Group 2 and 3 children are collected and recorded as part of the matching procedure from registry data provided by Statistics Sweden or via archival data from the original PATHS trial (for Group 1 children).

Etikprövningsmyndigheten
2022-05932-02-325884
2022-11-02

Overview of the child self-report surveys (completed in person with a research assistant)

Y1. Child's social emotional competence. The SSIS-SEL Brief survey, Student form (Anthony et al., 2020a) provides children's ratings of their own social emotional competence (SEC), namely their self-awareness, self-management, relationship skills, social awareness, and responsible decision making. This survey is consistent with the CASEL five competence domain model of social emotional learning (Weissberg et al., 2015) which is a guiding theory for this project. SSIS-SEL, Student consists of 20 items rated on a 4-point Likert scale with 0 (Never) to 3 (Almost Always) as the response options. A total and subscale scores provide an index of the child's SEC. This survey is designed to be used with children and adolescents (8 to 18-year olds) and has shown good psychometric properties in prior research studies, .90 Cronbach's alpha, test retest reliability = .87 (Elliott et al., 2020b).

Y2. SDQ. Child's mental health/well-being. The child assessment also includes the children's rating of their own mental health and well-being as indexed by youth reported edition of the Strengths and Difficulties Questionnaire (SDQ; Goodman, 1997). The SDQ has a total score and subscales for emotional symptoms, conduct problems, hyperactivity-inattention, peer problems and prosocial behavior. SDQ has 25 items rated on a 3-point Likert scale with response options that range from 0 = not true, 1 = somewhat true, 2 = certainly true. The SDQ has shown good psychometric properties in international and Swedish adolescent samples (e.g., .78 = Cronbach's alpha on the total SDQ score; Almroth et al., 2018).

Y3. SAQ. Child's school bonding. Children are asked to rating of their own experiences with school, on the General Adjustment subscale of the School Adjustment questionnaire. This five-item subscale rated on a 5-point Likert scale was developed for the FAST track study (CPPRG, 1997) and was used in a recent follow up the REDI trial cohort as adolescents (Sanders et al., 2020). This subscale has shown good reliability in U.S. based child and adolescent samples (e.g., .83 = Cronbach's alpha in Sanders et al., 2020).

Y4. Mental Health/Well-Being during C19, Youth. This study will be conducted during the COVID-19 pandemic, understanding how the pandemic maybe affecting the sample both children and parents, is a covarying historical factor important to consider and record in relation to our main project hypotheses and research questions. Although the effects of C19 are not a main study focus in this project, given that the study will be conducted in this time period, a short survey that has been used with adolescents in Sweden will be used in this study. This scale consists of nine items from the Experiences Related to COVID-19 instrument, which was developed by Skinner and Lansford (2020). This scale measures self-perceived changes in sleep, stress, satisfaction, loneliness, anger, depression, and anxiety, in connection with the pandemic. Items are measured on a 4-point scale (don't agree at all) to 4 (agree completely), and in a recent Swedish adolescent sample the scale showed good reliability (α = .82).

Table 2a. Child/Youth self-reported surveys/scales for Groups 1 and 2 and Time 1

Construct	Instrument	Subscales/Items Used	Scale Reference	Reliability (alpha)
**Social Emotional Competence**	**Y1.** SSIS-SEL Brief	20 items	Anthony et al. (2020a)	.90
**Social-emotional distress**	**Y2**. SDQ. Strengths and Difficulties Questionnaire (youth report)	25 items<br><br>***Sub scales***<br>Emotional symptoms<br>Conduct Problems<br>Hyperactivity-Inattention<br>Peer Problems<br>Prosocial Behavior	Almroth et al., 2018<br><br>Goodman, 1997; 2001	.78 (total score)
**School bonding**	**Y3**. SAQ. School Adjustment Questionnaire	5 items<br><br>General Adjustment general attitudes toward school	CPPRG, 1997<br><br>Sanders et al., 2020	.83<br>(in Sanders et al., 2020)
**Mental health/Well-Being during C-19**	**Y4**. C19. Experiences Related to COVID-19	10 items	Skinner & Lansford, 2020<br><br>Kapetanovic et al., 2021 COVIDung Project	.82<br>(in Kapetanovic et al., 2021)
**Total 60 items**				

Etikprövningsmyndigheten
2022-05932-02-325884
2022-11-02

Overview of the child performance tasks

Aspects of executive functioning (EF) will be assessed using several validated measures. The tasks described here are the constructs and tasks we select for use in this project. However, these listed tasks could be replaced by equivalent tasks (similar stimuli and do not pose any additional risk to participants), if the tasks described below prove to be too time consuming or if there are technical reasons that make their use not possible in this project.

Y5. Emotional Stroop task.

The Emotional Stroop task (Smolker et al., 2022) is a variation on the original Stroop task (Stroop, 1935). In the Emotional Stroop task, faces of adolescent with an emotion portrayed (happy or sad facial expression) are presented to participants. On top of the face, there is an emotional word written, such as the text “happy”. Participants are instructed to identify the emotional valance of the word (the text) and to disregard the facial expression that is shown along with the word. Participants use a left or right response key on a key board to indicate if a word is associated with a “bad” emotion or a “good” emotion respectively (Smolker et al., 2022).

The processes measured by the Stroop task are: internal control of behavior, processing speed (including emotional information processing), and inhibition.

This task requires the top-down internal control over behavior, which is required to select the stimulus-attribute on which behavior should be based (i.e., on the basis of the semantic meaning of the text of the word shown) rather than having the response being driven by salient distracting information (i.e., the face shown), and this indicates the ability to inhibit a prepotent response. The time needed to complete the Emotional Stroop task is approximately 10 minutes. Response time is measured in this task and accuracy in identifying the emotional valence of the text of the emotional word across trials. The Emotional Stroop showed good and theoretically expected convergent and discriminate validity with other cognitive constructs and indicators of adjustment in a sample of adolescents (N = 10, 796).

Etikprövningsmyndigheten
2022-05932-02-325884
2022-11-02

Y6. Hearts and Flowers Task. This task is also a computerized task that measures executive functioning including working memory, cognitive flexibility, and inhibitory control (Watts et al., 2018). This task has shown good psychometric properties in a longitudinal follow up study of a preschool social emotional competence intervention into adolescence in the U.S. called the Chicago school readiness project (Watts et al., 2018).

Specifically, the task presents children with either a heart or flower symbol that appears on either the left or right side of their computer screen. Children are asked to press the letter Q (left hand side of the screen and key board) when they see a Heart appear on the left side of their computer screen, and the letter P on the right-hand side of the keyboard and computer screen when they see a Heart appear on the right side of their computer screen. For the Heart symbol, when it appears on the computer screen, the location of the Heart is congruent with the location of the key on the keyboard that is pressed by the child, i.e., press on the same side as the stimulus. Q when displayed on the left, P when on the right, only when a Heart symbol appears on the computer screen.

When a flower appears on the computer screen, children are asked to do the opposite of what they are asked to do when a heart appears. When a flower appears, children are instructed to respond in the opposite way. When a flower appears on the left side the screen, children are asked to press the Q key which is on the right-hand side of the keyboard, and vice versa. For the Hearts and Flowers task, children are given practice trials, so they can show that they understand the instructions for the task.

The practice trials consist of a series of 12 hearts only (congruent) trials (randomly appearing on the left- or right-hand side of the screen), followed by 12 flowers only (incongruent) trials (randomly appearing on the left or right hand of the screen).

Etikprövningsmyndigheten
2022-05932-02-325884
2022-11-02

After the practice trials (which takes approximately 2 minutes in total), the actual task begins and this is a series of 33 symbols (which takes approximately 3 minutes in total), presented separately, that are a mix of hearts and flowers.

In the mixed trial, the number of correct responses divided by the total number of trials (i.e., accuracy) provide an index of working memory, cognitive flexibility and inhibitory control. Mean reaction time minus mean reaction time on the practice trials provides an index of cognitive demand. The task, scoring, and procedure were adapted from Watts et al. (2018). The time needed to complete the Hearts and Flowers task is approximately 5 minutes.

Y7. Sustained Attention to Response Task (SART)

The Sustained Attention to Response Task (SART; Robertson et al., 1997) is a performance task that presents on a screen 225 numbers, each in a random order, with the instruction to participants to press a response key except when shown the number 3; thereby providing an index of sustained attention (as indexed by attentional failures). SART has evidenced good psychometric properties in terms of test re-test reliability as well as concurrent and discriminant validity (Robertson et al., 1997).

Table 2b. Child/Youth performance tasks (Groups 1 and 2) at Time 1

Construct	Instrument	Time	Scale Reference
Internal control of behavior and inhibition	Emotional Stroop task	10 minutes	Smolker et al., 2022
Working memory, inhibition, and cognitive flexibility	Hearts and Flowers task	5 minutes	Watts et al., 2018
Attention	SART	5 minutes	Robertson, et al., 1997

Parent Assessment

The parent measures will be part of the follow up study (work package 1) and the intervention trial of the Parent Web (work package 2) for parents in Group 1. Parents other study arms (Groups 2 and 3) will have their assessment tailored to the role that the participant group has in the overall project. The parent assessment section here is broken down by study arm (group 1, 2, or 3) in order to provide clarity in regards to which parent assessment scales are given when and the study designs and work packages connected to the parent assessments.

Parent Assessment for Group 1 parents

Group 1 parents are involved in the complete project as a whole, in other words in work packages 1 and 2 (i.e., the follow up study and the trial of the Parent Web). Group 1 parents will be asked to provide demographic information about their participating child at study intake. According to the project procedure, study recruitment of Group 1 families occurs first and can take place via regular post (regular mail), phone (verbally with parents and researcher) or via consent form that is completed online.

Etikprövningsmyndigheten
2022-05932-02-325884
2022-11-02

Assessment for Group 1, G1T1 (Group 1, Time 1)

As participants are recruited assessments then take place on a rolling basis (G1T1 expected during Fall 2022). The parent assessment battery at this time point is relevant to the follow up study and also provides a pre-test assessment for the Parent Web intervention trial, for one parent in Group 1.

P1. Family Background Information. Parents are asked to provide background information about themselves (e.g., age, gender, socio economic status), family (language(s) preferred for use at home), and school situation for the participating child (9 questions, Wetterborg, Enebrink, et al. 2019).

P2. Parent rating of child's social emotional competence. The participating child's social emotional competence will be measured by the SSIS SEL Brief Scales-Parent (SSISb-Parent; Elliott et al., 2020a; Elliott et al., 2021). SSIS, Parent consists of 20 items rated on a 4-point Likert scale with 0 (Never) to 3 (Almost Always) as the response options. Total and subscale scores provide an index of the child's self-awareness, self-management, relationship skills, social awareness, and responsible decision making, which is theoretically consistent with the CASEL model (Weissberg et al., 2015). The instrument is designed for parents who have children from preschool through high school. The reliability and validity of the SSIS, Parent with U.S. parents is .91 Cronbach's alpha, test retest reliability = .88, and interrater reliability .61 for the total score (Elliott et al., 2020b). Validity evidence includes negative significant correlations with surveys of child emotional/behavioral problems (BASC-2) as well as positive significant correlations with surveys indexing adaptive behaviors (e.g., BASC-2 and Vineland II scales; Elliott et al., 2020b).

The constructs/scales P3 through P9 which are described below will be given to parents in Group 1 at G1T1 and at G1T2. The second parent assessment for Group 1 takes place during winter 2022/2023. The Time 2 assessment takes place as soon as Group 1 parents have completed the Parent Web. There is a 7-week approximate expected time frame to complete the Parent Web, thus we expect the Time 2 assessment to follow Time 1 assessment approximately 7 weeks later but the exact timing will depend on when parents in Group 1 start and take part in the Parent Web.

As we only have one parent take part in the follow up study (i.e., work package 1), if a parent in Group 1 who was not in the follow up study would like to participate in the Parent Web (i.e., work package 2) the assessment for Group 3 Time 1 will be given to Group 1 parents who have not participated earlier in this project and this serves as a pre-test assessment for the Parent Web.

P3. SDQ. Parent rating of child's mental health/well-being. The parent rating of the participating child's emotional problems, conduct problems, hyperactivity, peer problems and prosocial behavior are indexed by the Strengths and Difficulties Questionnaire (SDQ; Goodman, 1997; Malmberg et al., 2003). The SDQ consists of 25 items rated on a 3-point Likert scale with response options that range from 0 = not true, 1 = somewhat true, 2 = certainly true. Ordinal alphas for SDQ subscale scores in a Swedish parent sample ranged from .85 to .91 (Björnsdotter et al., 2013).

WP2 Parent Web constructs/scales for parents at G1T1 and G1T2
(Group 1 at Times 1 and 2).

Measurement of Primary Outcomes in the Parent Web trial (WP2)

Based prior research about the Parent Web (Wetterborg, Enebrink, et al. 2019), we expect that the ***primary outcomes*** targeted for intervention change will be reductions in youth externalizing problems and family conflict as well as increased warmth in families, as rated by parents.

Parent rated youth externalizing problems.

P3. SDQ, Conduct Problems Scale. Relevant items/scale, namely the conduct problems subscale on the Strengths and Difficulties Questionnaire (SDQ; Goodman, 2001). This subscale of the SDQ has shown borderline to good reliability (i.e., Cronbach's alpha ranging from .57 to .67 in Wetterborg, Enebrink et al., 2019, and ordinal alpha was .89 for this scale in Bjornsdotter et al., 2013).

P4. DBD, Oppositional Defiant Scale of the Disruptive Disorder Rating Scale consists of 7 items related to parents view of their child's behavioral problems/defiant behavior (DBD; Pelham, et al., 1992). DBD questions are rated on a 4-point scale ranging from 1 (not at all) to 4 (very much). The instrument has shown good reliability (i.e., Cronbach's alpha ranging from .86 to .90 in Wetterborg, Enebrink, et al., 2019).

Parent rated family warmth and conflict

P5. FCU. Warmth and conflict are indexed by eight items rated on a five-point scale from 1 (never) to 5 (more than 7 times) with items from diverse scales [e.g., FCU Caregiver Assessment Scale obtained from the Adult-Child Relationship Scale (ACRS; Criss, & Shaw 2005) from project PAL 2 in the Child and Family Center, University of Oregon, Eugene] as used in Wetterborg, Enebrink et al. (2019). Five items measure parent-child warmth and three items measure parent-child conflicts. Scale alphas were good and ranged from .74 to .85 for these scales in Wetterborg, Enebrink et al. (2019).

Measurement of Secondary Outcomes in the Parent Web trial (WP2)

Secondary intervention outcomes concern hypothesized intervention related declines in youth emotional symptoms and peer problems, as well as increased prosocial behavior as indexed by relevant items/scales on the SDQ. Other secondary outcomes include increased use of positive parenting practices as measured by the Parenting Children and Adolescents Scale (PARCA) as well as reductions in parents' stress (Perceived Stress Scale; Cohen et al., 1983) and anxiety and depression (Hospital Anxiety and Depression Scale; Zigmond & Snaith, 1983) and improvements in parents' acceptance of their children's emotion displays as measured by the Tolerating subscale of the Affective Style Questionnaire (Hofmann & Kashdan, 2010).

P6. PARCA, *Parenting Children and Adolescents Scale* (Ringle et al., 2019). The PARCA has 19 items rated on a five-point scale and has shown with good psychometric properties (Ringle et al. 2019). The scale has three subscales on encouragement of positive behaviors, setting limits, and proactive parenting behaviors. Scale alphas were good and ranged from .86 to .91 for these scales in Ringle et al. (2019).

P7. PSS, Parental stress. The Perceived Stress Scale (PSS; Cohen, Kamarck, & Mermelstein, 1983) is a frequently used scale with 10 questions about perceived stress during the last month rated on a 5-point Likert scale. The scale measures the extent to which situations in one's life are assessed and perceived as stressful and current stress levels. Good validity has been shown for the instrument. Scale alphas were good and ranged from .83 to .86 for this scale in Wetterborg, Enebrink et al. (2019).

P8. HAD, Parental Health. Prevalence of Depression and Anxiety in Parents: Measured by Hospital Anxiety and Depression Scale (HAD; Zigmond & Snaith, 1983). HAD consists of two scales (a total of 14 items), one for anxiety and one for depression with seven questions each. The instrument is well validated and has been used in large studies both in Sweden and internationally (Bodlund, 1997). Scale alphas were good and ranged from .70 to .81 for these scales in Wetterborg, Enebrink et al. (2019).

Etikprövningsmyndigheten
2022-05932-02-325884
2022-11-02

P9. ASQ, Affective Style. Tolerating subscale of the Affective Style Questionnaire (***ASQ***; Hofmann & Kashdan, 2010) consists of five items that examine how a parent is doing in regards to validating their child's emotions when expressed. Scale alphas were good and ranged from .80 to .89 for these scales in Wetterbor g, Enebrink et al. (2019).

Covariate

P10. Mental Health/Well-Being during C19, Parent. This study will be conducted during the COVID-19 pandemic, understanding how the pandemic maybe affecting the sample both children and parents, is an important historical factor to consider within our main project hypotheses and research questions. Like the child edition of this scale, the adult edition is identical and was developed by Skinner and Lansford (2020).

Intervention Implementation Parent Web

P11. Implementation/Consumer Satisfaction Survey. Parents' satisfaction with the Parent Web is measured by questions about each module that appear directly after each module is completed, with a response of how useful a session has been, how videos, information are perceived by parents (7 questions; Wetterborg et al., 2019), other information about number of lessons viewed are recorded as parents complete the Parent Web modules. Information that is collected to measure intervention implementation are listed in the table below, only parents taking part in the Parent Web provide this information, i.e., parents in Groups 1 and 3.

Table 3. Intervention Implementation as reported by Parents during Parent Web Intervention

**Construct**	**Instrument**	**Subscales/Items Used**	**Scale Reference**
Parents' satisfaction with the Parents' Web, questions on utility of PW, satisfaction with PW materials etc.,	Consumer Satisfaction of the Program Survey	7 questions embedded in PW modules	Wetterborg et al. (2019)
Parents' lesson coverage (LC) module/lesson completion		embedded in PW modules	Wetterborg et al. (2019)
Time spent in modules (D) intervention dosage		embedded in PW modules	Wetterborg et al. (2019)

Etikprövningsmyndigheten
2022-05932-02-325884
2022-11-02

Parent Assessment for all Parent Groups 1, 2, and 3.
The parent assessment at each time point for the respective study arms is listed below in a series of tables by group and by time point. See Table 4.

Table 4a. Parent Report Survey/Scales Group 1, Time 1

Construct	Instrument	Items	Scale Reference	Reliability<br><br>Alpha
**Parent's view of their child**				
Background information about self and family	**P1. PD**<br><br>Parent Demographics	9 questions	Wetterborg, Enebrink, et al. 2019).	Not applicable
Child's social emotional competence (rated by parents)	**P2. SSIS-SEL Brief Parent**	20 questions	Elliott et al., 2021; Elliott et al., 2020a	.91
Child's<br><br>Social-emotional distress	**P3. SDQ**<br><br>Strengths and Difficulties Questionnaire SDQ (parent report)	25 questions	Björnsdotter et al., 2013<br><br>Goodman, 1997; 2001	.85 to .91<br><br>Björnsdotter et al., 2013
Children's behavioral problems /defiant behaviour	**P4. DBD**<br><br>Disruptive Behavior and Disorder scale (DBD)	7 questions	Pelham, et al., 1992<br><br>Wetterborg, Enebrink, et al. 2019	.86 to .90<br><br>Wetterborg, Enebrink, et al. 2019
**Parent's view of their family, themselves, and their parenting**				
Warmth and conflict in the family	**P5. FCU**<br><br>Caregiver Assessment Scale obtained from the Adult-Child Relationship Scale	8 questions	Criss, & Shaw 2005<br><br>Wetterborg, Enebrink, et al. 2019	.74 to .85<br><br>Wetterborg, Enebrink, et al. 2019
Parenting strategies/ behaviors	**P6.** PARCA, Parenting Children and Adolescents Scale	19 questions	Ringle et al. 2019	.86 to .91<br><br>Ringle et al. 2019

Etikprövningsmyndigheten
2022-05932-02-325884
2022-11-02

Table 4a. Parent Report Survey/Scales Group 1, Time 1 (continued)

Construct	Instrument	Items	Scale Reference	Reliability<br><br>Alpha
Parental stress	**P7. PSS** Perceived Stress Scale (PSS)	10 questions	Cohen, Kamarck, & Mermelstein, 1983<br><br>Wetterborg, Enebrink, et al. 2019	.83 to .86<br><br>Wetterborg, Enebrink, et al. 2019
Parental Health	**P8. HAD** Hospital Anxiety and Depression Scale (HAD)	14 questions	Zigmond & Snaith, 1983<br><br>Wetterborg, Enebrink, et al. 2019	.70 to .81<br><br>Wetterborg, Enebrink, et al. 2019
Validation	**P9. ASQ** Affective Style Questionnaire	5 questions	Hofmann & Kashdan, 2010	.80 to .89<br><br>Wetterborg, Enebrink, et al. 2019
Mental health/Well-Being during C-19	**P10. C19P**<br><br>Experiences Related to COVID-19	10 items	Skinner & Lansford, 2020<br><br>Kapetanovic et al., 2021 COVIDung Project	.82<br><br>(in Kapetanovic et al., 2021)
**Total Parent reported items**<br><br>**127 questions**				

Etikprövningsmyndigheten
2022-05932-02-325884
2022-11-02

Table 4b. Parent Report Survey/Scales Group 1, Time 2

**Construct**	**Instrument**	**Items**
**Parent's view of their child**		
Child's<br>Social-emotional distress	**P3. SDQ**<br>Strengths and Difficulties Questionnaire SDQ (parent report)	25 questions
Children's behavioral problems /defiant behaviour	**P4. DBD**<br>Disruptive Behavior and Disorder scale (DBD)	7 questions
**Parent's view of their family, themselves, and their parenting**		
Warmth and conflict in the family	**P5. FCU**<br>Caregiver Assessment Scale obtained from the Adult-Child Relationship Scale	8 questions
Parenting strategies/ behaviors	**P6.** PARCA, Parenting Children and Adolescents Scale	19 questions
Parental stress	**P7. PSS** Perceived Stress Scale (PSS)	10 questions
Parental Health	**P8. HAD** Hospital Anxiety and Depression Scale (HAD)	14 questions
Validation	**P9. ASQ** Affective Style Questionnaire	5 questions
**Total Parent reported items**<br>**88 questions**		

Etikprövningsmyndigheten
2022-05932-02-325884
2022-11-02

Table 4c. Parent Report Survey/Scales Group 2, Time 1

**Construct**	**Instrument**	**Items**
**Parent's view of their child**		
Background information about self and family	**P1. PD**<br>Parent Demographics	9 questions
Child's social emotional competence (rated by parents)	**P2. SSIS-SEL Brief Parent**	20 questions
Child's<br>Social-emotional distress	**P3. SDQ**<br>Strengths and Difficulties Questionnaire SDQ (parent report)	25 questions
Mental health/Well-Being during C-19	**P10. C19Parent**	10 questions
**Total Parent reported items**<br>**64 questions**		

Etikprövningsmyndigheten
2022-05932-02-325884
2022-11-02

Table 4d. Parent Report Survey/Scales Group 3, Time 1

**Construct**	**Instrument**	**Items**
**Parent's view of their child**		
Background information about self and family	**P1. PD**<br><br>Parent Demographics	9 questions
Child's<br><br>Social-emotional distress	**P3. SDQ**<br><br>Strengths and Difficulties Questionnaire SDQ (parent report)	25 questions
Children's behavioral problems /defiant behaviour	**P4. DBD**<br><br>Disruptive Behavior and Disorder scale (DBD)	7 questions
**Parent's view of their family, themselves, and their parenting**		
Warmth and conflict in the family	**P5. FCU**<br><br>Caregiver Assessment Scale obtained from the Adult-Child Relationship Scale	8 questions
Parenting strategies/ behaviors	**P6.** PARCA, Parenting Children and Adolescents Scale	19 questions
Parental stress	**P7. PSS** Perceived Stress Scale (PSS)	10 questions
Parental Health	**P8. HAD** Hospital Anxiety and Depression Scale (HAD)	14 questions
Validation	**P9. ASQ** Affective Style Questionnaire	5 questions
Mental health/Well-Being during C-19	**P10. C19P**<br><br>Experiences Related to COVID-19	10 items
**Total Parent reported items**<br><br>**107 questions**		

Etikprövningsmyndigheten
2022-05932-02-325884
2022-11-02

Table 4e. Parent Report Survey/Scales Group 3, Time 2 and Time 3

**Construct**	**Instrument**	**Items**
**Parent's view of their child**		
Child's<br>Social-emotional distress	**P3. SDQ**<br>Strengths and Difficulties Questionnaire SDQ (parent report)	25 questions
Children's behavioral problems /defiant behaviour	**P4. DBD**<br>Disruptive Behavior and Disorder scale (DBD)	7 questions
**Parent's view of their family, themselves, and their parenting**		
Warmth and conflict in the family	**P5. FCU**<br>Caregiver Assessment Scale obtained from the Adult-Child Relationship Scale	8 questions
Parenting strategies/ behaviors	**P6.** PARCA, Parenting Children and Adolescents Scale	19 questions
Parental stress	**P7. PSS** Perceived Stress Scale (PSS)	10 questions
Parental Health	**P8. HAD** Hospital Anxiety and Depression Scale (HAD)	14 questions
Validation	**P9. ASQ** Affective Style Questionnaire	5 questions
**Total Parent reported items**<br>**88 questions**		

Etikprövningsmyndigheten
2022-05932-02-325884
2022-11-02

Etikprövningsmyndigheten
2022-05932-02-325884
2022-11-02

Neighborhood assessment and SSO procedure

The systematic social observation (SSO) procedure and rating form items that are used in this project were derived from previous studies (e.g., Odgers et al., 2009, 2012; Quinn et al., 2016). We have refined our SSO procedure and items based on a pilot study that was conducted as part of doctoral project connected with the original PATHS intervention trial in Sweden. In the doctoral study conducted by Ingela Clausén Gull, the SSO methods (in person and virtual via Google street view) were used to measure neighborhood factors in the immediate neighborhood surrounding the preschools in the PATHS intervention trial. In this ongoing PhD., project studies will triangulate ratings from the SSO method (on site and virtual) with other related methods (e.g., registry data on local inhabitants' income and highest education at the postal code level, and an anonymous resident survey in the same postal codes). We will use an abbreviated edition of SSO protocol/observation rating procedure to measure neighborhood factors for participants' in Groups 1 and 2 at Time 1.

Participating children in Group 1 and 2 are matched on individual demographic factors (e.g., gender, age) and these children will be living in the same postal codes (another one of the matching criteria).

The neighborhood assessment is specifically related to purpose 2, hypothesis 3 which are listed below:

> **PURPOSE 2:** To identify the ways in which the socio-economic conditions of the neighborhoods that children live in are important to children's social emotional competence and mental health.
>
> *Hypothesis 3*: Across cohorts (in the original PATHS cohort and matched sample, Groups 1 and 2), elevated social emotional competence (SEC) in children will buffer the effects of contextual risks at the neighborhood level, and will be associated with better mental health.

SSO assessment. Virtual classification of the participating children's local neighborhood environment will be conducted with the SSO protocol rating form which focuses on the presence of physical factors such as the presence of graffiti and vandalism, availability of green spaces and type of buildings and infrastructure. Specifically, the SSO protocol that is used in this project consists of 37 questions that raters respond to while virtually observing streets in the vicinity of participants' homes. Response options are either yes or no or on a Likert scale, specific constructs measured are physical disorder (4 items), availability of recreation (2 items), types of residences in the area (7 items), Condition of Signs and Communication (3 items), availability of businesses and services (13 items), and overall condition of the area (5 items).

In a recently completed pilot study, in areas where the PATHS preschools are located, inter rater reliability of two raters for both in person (observation conducted in real life, in person) and virtual ratings of the same streets using Google Street View were .60 or higher as measured by kappa or intra class correlations (ICCs) or .70 and higher if measured by percentage of agreement between the two independent raters. On the rating form, there are also 12 additional items that are for measurement quality control by noting the timing of the observation, image quality, etc., and thus reliability ratings between observers are not relevant for those 12 items.

Detailed Procedure for SSO assessment

The virtual classification will be made by a trained rater/observer who is a member of the research team. The area to be rated will be a random selection of three to four 800m areas within the postal code that participating children live in. Natural boundary lines (such as major roads, streams, railways) will be considered in setting this radius for each of the three to four areas to be observed and rated. The three to four areas in each postal code will be picked randomly by a research team member who is not the rater/observer, however an exclusion criterion for a randomly selected area is that the area should not contain any of our participants' homes (this applies to participants in Groups 1, 2, or

Etikprövningsmyndigheten
2022-05932-02-325884
2022-11-02

3). To protect the participants' privacy, we will not view any participant's home as part of the virtual observation through Google Street View. The areas to be viewed will be randomly selected at the postal code level and will not include participants' homes, but be in the local vicinity (i.e., within the postal code) that children in Groups 1, 2, and 3 live in. SSO assessment is focused on analyses that involve Groups 1 and 2. However, because of the matching procedure, families in Group 3 will also live in the postal codes that the SSO assessment will take place. No SSO analysis will be used in any analyses that involve Group 3.

The randomly selected three to four areas per postal code will be viewed through Google Street View by the trained observer/rater. In person (in real life) ratings/observations will only be carried out for an area, if the virtual images taken at ground level in Google Street View do not provide sufficient information or image quality for a valid rating to be made.

To better ensure the reliability and validity of our SSO protocol, we will have two raters independently rate a subset of the sample of areas in the wider pool of areas to examine inter rater reliability in this project is acceptable. And the concurrent validity of the SSO assessment will be tested by comparing the SSO ratings for areas within postal codes to registry data of the income and highest education of inhabitants in the selected postal codes. This registry data at the postal code level for inhabitants' highest education and income is available and will be provided by Statistics Sweden. This comparison between SSO and registry data will be examined through a cross-sectional research design (one time point) with correlational or other analyses. For example, it could be expected that positively rated neighborhood features (e.g., availability of green spaces) would be positively and significantly related to higher community level (all inhabitants in a postal code) education and income.

If the SSO methods show acceptable reliability and validity in this project, these neighborhood level factors will be connected to child level assessments of children's social emotional competence and mental health, in analyses that examine hypothesis 3.

Registry data at the postal code level

We will request from Statistics Sweden that they extract registry data for the postcode areas relevant to participants in Groups 1 and 2 (e.g., postal code in which children's homes are located). The extracted register data is deidentified and it is not possible to link to any particular individual, the information provided from the registry is monthly income and highest education of all inhabitants in a postal code which is reported at the group level. The supplied data files will only be available to the researchers working on the study. The files are stored electronically in an encrypted cloud service or on password-protected computers in a local, closed computer system at the Department of Psychology. In order for researchers to be able to access the files, a user identity and personal password are required.

Etikprövningsmyndigheten
2022-05932-02-325884
2022-11-02

Universal Parent Education, the Parent Web (Work Package 2)

The Parent Web (PW) is an internet delivered, Swedish developed educational training program for parents of older children through adolescents (11–17-year olds). PW is designed to support parenting practices and parent-adolescent interactions that encourage prosocial behavior and emotional regulation in youth, reduce instances of coercive parenting that encourage aversive, negative escalatory interactions in families, improve assertive communication and problem solving in families as well as increase warmth and closeness among parents and adolescents. PW is available in universal and selective versions, both of which are rooted in social learning and coercion theory. The selected edition of PW (i.e., parents perceived they were having many conflicts with their children) was first designed and tested with promising results (Lönn-Rhodin, Forster, & Enebrink, 2015).

A universal edition of PW was also developed and this is the edition of the educational training program is evaluated in this project (i.e., the main focus of work package 2). The universal edition of PW consists of five basic modules including an introductory module (one basic module completed per week, typically across seven weeks) and informational bonus modules on mental health, school, the Internet, friends, bullying, alcohol and drugs are unlocked after the basic modules are completed. Basic modules contain information, exercises and homework, and videos of actors demonstrating ideas and parenting practices in role play vignettes. Across modules, emphasis is on increased use of positive parenting, namely emphasizing the value of time spent together, emotional validation, communication, and problem solving. A family guide, who is a trained research assistant, supports parents as they work through the modules. The PW modules (universal edition) are listed in the table below.

Table 5. Content of the Parent Web (universal edition)

Welcome
Introduction
Section 1 - Time together
Section 2 - Listen and confirm
Section 3 - Choosing battles and solving problems
Section 4 - Letting Go and Staying
Bonus episode - Mental illness
Bonus episode - Help at home
Bonus section - School
Bonus episode - Friends and bullying
Bonus section - Internet and games
Bonus section - Alcohol and drugs

Etikprövningsmyndigheten
2022-05932-02-325884
2022-11-02

Parent Web (PW) Outcomes

Our expectations regarding the changes related to or outcomes of PW are derived from the selected intervention trial of PW by Wetterborg, Enebrink, et al. (2019). We hypothesize that the primary outcomes of PW will be improved parental well-being (e.g., Brief Quality of Life Scale), family warmth and reduced conflicts (items from Wetterborg et al.), increased use of positive parenting practices, improvements in supervision and consistent parenting (PARCA Scale); reductions in parents' stress; anxiety and depression (Hospital Anxiety and Depression Scale); improvements in parents' acceptance of children's emotions (Tolerating subscale of the Affective Style Questionnaire). Secondary outcomes will be reduced youth externalizing problems and increased prosocial behavior (SDQ). We will pre-register the PW trial protocol prior to data collection.

Sample sizes in the work packages

The sample in each study arm is based on the original Swedish PATHS intervention trial with an immediate intervention group of up to 144 children. In work package 1 (i.e., the PATHS follow up study), we expect up to 288 children to actively participate in the project, with up to 144 children in each one of the two study arms, namely children in Groups 1 (the PATHS cohort) and 2 (matched comparison children).

For parents, we expect to invite one parent per participating child to take part in work package 1, this would equate to a maximum of 288 parents participating in work package 1. Parents in Group 1 will also take part in work package 2. Only parents in Group 3 will be invited to take part in work package 2 (the Parent Web trial). For work package 2, both parents can participate and this would equate to a maximum of 576 parents in total, with 288 parents in Group 1 and 288 parents in Group 3.

Regarding statistical power, for a contrast of means between two independent groups and an effect size corresponding to Cohen's definition of a medium effect (i.e., a standardized path coefficient (β) of .30), sample size needed to achieve power of .80 is 64 per group. The trial sample size will meet this criterion and will also be guided by these power considerations for planned intervention moderation analyses, as well as group comparisons conducted in descriptive study analyses in work package 1.

Study designs and intervention allocation

There are 3 main research designs in this project:

1. Cross sectional. The follow up of the PATHS cohort (Group 1) relative to a matched comparison group, both groups at age 11 to 13 years old, Group 1 and 2. Work package 1, hypothesis 1, 2, and 3.

2. Longitudinal. Within group analysis of the PATHS cohort (Group 1 only) from age 4-5 to 11-13, which calls for a longitudinal study design. Work package 1, hypothesis 2. The 4-5 year old data is archival from the original PATHS trial.

3. Quasi-experimental trial with pre and post-testing. The evaluation of the Parent Web intervention involves PATHS cohort children's parents (Group 1) relative to a wait-list control group of parents (Group 3) which calls for a quasi-experimental wait list control design with a pre and post-testing. Group 3 parents are on a wait-list and thus have a pre and post with no PW exposure, followed by participation in the PW that is followed by an additional post-test (3 assessment time points). Group 1 parents have a pre-test (which is integrated into the follow up assessment in work package 1) and participation in PW followed by a post-test (2 assessment time points). These assessments are relevant

to Work package 2, hypothesis 4, research question 1 and 2. In regards to intervention allocation in the PW trial, the assignment to intervention condition is not random, given that the project is designed as a follow up of a completed intervention trial with a booster intervention for a particular group of parents (i.e., families).

Statistical methods

There are several statistical analyses involved in the project hypotheses and research questions, and the main analysis plans are described here.

Work package 1, main analysis plan

We expect to examine hypothesis 1 and 3 by using structural equation models such as that shown in Figure 1. In this model, paths A-B represent the hypothesized effects of participating in the PATHS intervention as 4- or 5-year olds on social emotional competence (path A) and mental health (path B) at age 11 to 13 years old. To compare PATHS children (Group 1) with a matched comparison group of children (Group 2), a binary group contrast variable will be created to represent the groups (1 = PATHS, 0 = matched comparison, group 2 children). When possible, social emotional competence and mental health constructs will be modelled as latent variables (the circles in Figure 1) with either item or parcel indicators. Children and parents report on the child's social emotional competence and mental health/well-being. Model analyses will take place in Mplus or R. We expect that these analyses will use a robust maximum likelihood estimator to account for non-normally distributed data and a Huber-White adjustment to standard errors to account for the clustered nature of the data at the neighborhood level. Full information maximum likelihood will be used to address missing data.

The analysis plan for Hypothesis 2 involves the estimation of the value of changes in SEC, and this analysis will be primarily based on probability modeling and follow-up data on PATHS children's outcomes in terms of SEC, behavioral adjustment, and mental health. An exhaustive literature review will be conducted in order to identify linkage studies for modeling of developmental trajectories. These empirically-supported probability-based changes in behavior over time will then be valued.

Etikprövningsmyndigheten
2022-05932-02-325884
2022-11-02

Etikprövningsmyndigheten
2022-05932-02-325884
2022-11-02

Figure 1. Model to test hypothesis 1 and 3.

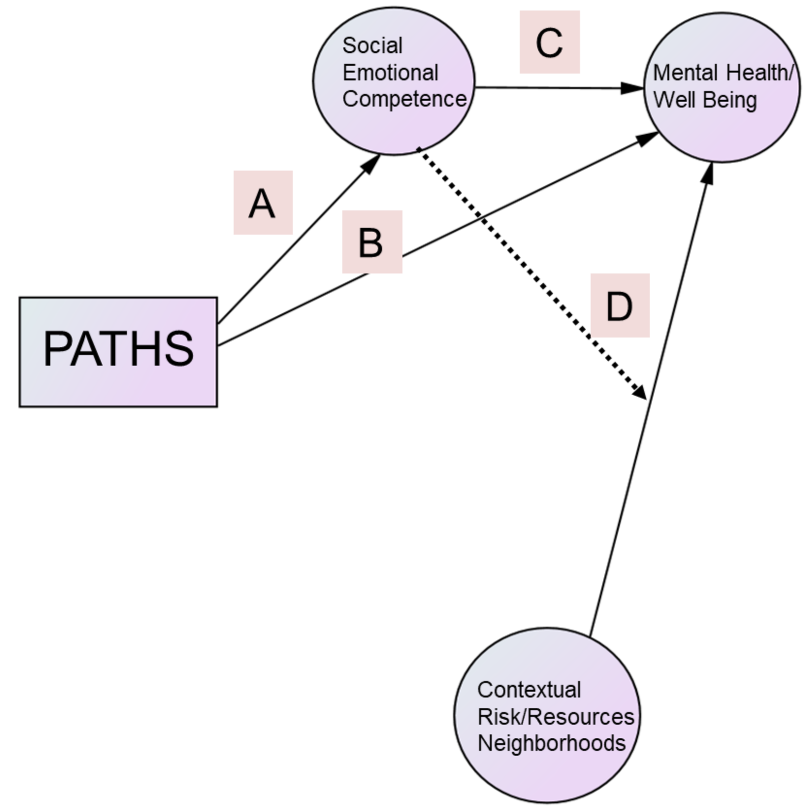

Figure 3. Conceptual model for the cross-sectional analyses follow up of PATHS cohort children (Group 1) relative to matched comparison children (Group 2), child and parent ratings of children included in this model. This model is relevant to work package 1.

Work package 2, main analysis plan

Hypothesis 4 will be tested for each outcome construct by estimating two-wave latent growth models. In a two-wave latent growth model, the latent intercept factor provides an estimate of the mean initial score, while the latent slope factor provides an estimate of the mean change score. When covariates of the slope are included, the path coefficient for any particular covariate provides an estimate of the effect of that covariate on change in the outcome variable, holding constant all other covariates. This type of model is analogous to ANOVA but allows adjustment of latent intercepts and latent slopes for measurement error. To compare groups, a binary group contrast variable will be created to represent the groups (e.g., Condition 1 will be coded 1 = original PATHS cohort children's parents, 0 = matched sample, group 3 parents). The contrast variable will be added to a two-wave latent growth model as covariates of the slope factor.

Expected outcomes of the project

Scientific outcomes of the project promise new insights and innovations. For example, in the area of social emotional learning (SEL). SEL interventions like PATHS aim to directly support the growth of emotion regulation. Globally, only two prior studies have had a more than a four year follow up of a preschool SEL intervention (e.g., Bierman et al., AJP advance). A six to eight year follow up, as will be conducted in this project (work package 1), has never been conducted outside of the U.S. Beyond documenting the long term impact of working systematically to support young children's social emotional competence in preschool, this project also advances knowledge about developmental trajectories relevant to emotion regulation (which is a key aspect of social emotional competence) and mental health, which can inform emotion regulation and wider social emotional learning interventions (Moltrecht et al 2020).

Also in regards to the booster intervention, scientifically the Parent Web (PW) adds value in that evidence-based parent training interventions in general are clearly connected to improved mental health and well-being in children and parents. Past research on the effect of the selected edition of PW are promising, showing benefits in reduced externalizing problems in youth (as rated by parents) as well as less depression and stress in parents.

If the results of this trial at the universal level, and other trials of PW prove promising, this then lays the ground for more evidence-based options for universal parent training for families with adolescents, and connected benefits in improved mental health and well-being for the participants in this study as well as for other families that might benefit from this type of intervention. PW also connects with parents online. A beneficial parent training program that is easily accessible through the internet represents an ideal type of intervention, in light of the C19 pandemic and is well suited to after the pandemic, when demand for more flexibility via digital services will remain important to addressing health inequalities.

Finally, also from a scientific standpoint, the development of the SSO methods in this project are innovative and more knowledge is needed about how local neighborhood factors (e.g., risks/strengths in the built environment) relate child and adolescent development and the prevalence of mental illness. Understanding neighborhood risk and its connections to child SEC and how the promotion of such competencies may buffer risk in Swedish urban settings would is valuable in a number of respects including informing the design of future coordinated multi-level (i.e., person-context) interventions.

In summary, this project promises several direct benefits to study participants, such as helping to have a voice in and directly contributing to what is known about youth development, family life and mental health in a contemporary, Scandinavian context. Participation in work package 2, also is designed so

Etikprövningsmyndigheten
2022-05932-02-325884
2022-11-02

that parents are better supported during their child's transition into adolescence, which could thereby have beneficial effects on children/youth participating in this study (relevant to Groups 1 and 3).

Dissemination of results

We expect to report project findings in open access scientific publications (e.g., peer reviewed). We also plan to create annual project updates distributed through our project website and to engage in outreach about our study and the topics that are involved in the project (via meetings, a project forum event with community stakeholders). Study results will not be described until all relevant project activities are completed. All results are reported at a group level.

Ethics and Data Safety

This project will follow Swedish law and relevant GDPR requirements. All study participation is voluntary with parental informed consent required for participating children and consent for adult study participants. Participating children also provide written consent for study participation. In the study procedures children at the time of assessment with again be informed about the voluntary nature of study participation and that they have the right to stop participation in any project activity at any time without any adverse consequences. Written parental and child consent will take place prior to data collection. All project results are reported at the group level and not at an individual study participant level.

Etikprövningsmyndigheten
2022-05932-02-325884
2022-11-02

All data collected in this study are one of the following: child and parent survey responses to standardized surveys widely used in the child and adolescent research literature, ratings of neighborhood features that are observer reported. The observer is a trained research assistant. Other data collected in this project is anonymous registry data on educational background and income at the postal code level for all participants' living in a particular postal code. There are no audio or video recordings collected for this project.

All parent assessment/data collection takes place online, and parents' participation in the Parent Web will also be online. All online project activities (e.g., consent forms, parent assessments, intervention participation by parents) will use a secure platform for data safety has 2-factor authentication and is located at Karolinska Institute. The data collection/ management system to be used in this project is already in use by Dr. Enebrink as part of other ongoing studies that evaluate the effects of online social and behavioral interventions. This online platform will be expanded to fit the relevant parts of this project (e.g., parent assessment and parents' participation in the Parent Web). Data from this platform in regards to this study will be stored electronically in an encrypted cloud service managed by KI. In the data platform, project identification numbers are assigned instead of the use of personally identifying information.

All child assessment/data collection takes place online or in person. Online or in person assessment with child participants is needed given the age of the children who are 11 to 13 years old, particularly in regards to the performance tasks on executive functioning. Administration of these tasks requires a trained adult/researcher to be present so that children understand the task before they perform it and can ask questions about how the task works, if they have such questions.

Assessment with adolescents in Groups 1 and 2 will take place on a mutually agreed upon day, time and location (i.e., agreed upon by both parents, the child, and research team). Locations for the child assessment include the child's home (in real life) or online assessment from home (child is at home, researcher is available online via online meeting platform), with one or more parents present when the assessment occurs. And the executive functioning tasks will be computerized.

If the C19 pandemic is still a public health concern at the time of the child assessment (assessment expected to be in Fall 2022), then research assistants who are collecting the child assessments will wear a medical mask during the child assessment. Parents and children will be informed of the C19 precautions that the research team will take prior to the child assessment. Families who do not want to have an in-person child assessment will be offered an online child assessment, the procedure for the online child assessment option will follow the procedures for the parent assessment.

How does the risk and benefit balance change with the proposed changes in the ethics addendum submitted on Nov. 2, 2022?

Change 1:

We evaluate that the increased number of families in groups 2 and 3 does not change the risk/benefit balance for the study, and only provides a sounder scientific test of the conclusions to be drawn from the study results.

Change 2:
Potential risks with online assessment are minor and we will take several steps to minimize potential risks. The benefits of change 2 include more accessibility and convenience for child participants to take part in the project. In regards to possible risks, one risk may potentially include children being disturbed while doing the assessment or that children will have difficulties understanding the tasks. For that reason, we will request parents to agree on a time slot for child assessment, when the child can sit relatively undisturbed, in front of a computer. Before, during and after each child assessment a trained researcher/research assistant will be present online via zoom to ensure that children understand both their rights as the participants, as well as the online surveys and cognitive tasks.

Where online data from child assessments is stored: Data from the child survey will be stored electronically in an encrypted cloud service managed by KI (same platform as the parent surveys), this part of the project has already been approved in the original ethics approval for this project. Data from the cognitive tasks will be downloaded on a daily basis into a secure encrypted database located at Stockholm University at the Department of Psychology that is managed by the research team. The cognitive task data is first collected by the cognitive task company in a GDPR compliant, secure cloud service with the server located in the EU. Data can then be removed from the company cloud and downloaded to a data cloud at Stockholm University, the Stockholm University data cloud is encrypted and GDPR compliant (Sunet Drive).

Online assessments with children. The zoom video platform is encrypted and supported for use by Stockholm University for various purposes online courses, meetings, and research projects. When having the child assessment via zoom, the research team will take several steps to ensure greater privacy and security. We will use the edition of zoom purchased by Stockholm University via the Sunet/NORDUnet which is consistent with GDPR and other European data protection laws and all data is stored in the EU when using the university paid edition of zoom. For all zoom meetings with children, we will create a meeting with a unique password so that only those with the password can sign on to the zoom meeting. And the team will regularly (on a weekly basis) update zoom so all security updates are current. There will be no audio or video recordings of participants during the zoom meeting, the purpose of the zoom meeting is only to explain instructions for the child survey and cognitive task and to be present with the child if the child has any questions. If children or parents do not wish to have the child assessment online, for any reason, they will be offered to do the child assessment with an in-person home visit that follows the procedures in the original ethics approval for this project

Etikprövningsmyndigheten
2022-05932-02-325884
2022-11-02

In general, all data collected from participants in this project are confidential and the link that allows for identification of an individual participant's data will be stored separately from the collected data. Only the main study administrators will have access to this linking information, which will be stored in either a fireproof safe (paper form) or password protected encrypted database (electronic form). Project data and the link that allows for identification of an individual participant's data will be stored indefinitely in the Stockholm University data archives.

Participation in this project is not expected to result in adverse consequences to the study participants. Although we use the term intervention to describe the Parent Web, it is a universal (i.e., designed for all families) parent training education program that provides information and promotes skills that are suitable to parents in general. However, because mental health information is collected as part of the study, the clinical psychologist team members (e.g., Dr. Johanna Stålnache and Dr. Pia Enebrink) will monitor for adverse events and facilitate service referral (e.g. social/mental health) as needed. Parents are informed about the need for referral, if the service referral involves child/youth study participants.

Data access

Data analyses will be primarily conducted by the research team members in Sweden. We except some collaboration on data analyses and reporting of study results with one expert outside of Sweden. Specifically, we have arranged for project data to be analyzed by a member of our research team that resides in the United States: Dr. Kyle Eichas at Tarleton State University, Waco Texas, USA. All transfers of data to Dr. Eichas will be conducted with de-identified data that are encrypted and transferred via a secure cloud server that is located in Sweden. While data analyses are conducted by Dr. Eichas the edition of the data that he is working with will be stored on an encrypted external hard drive that will be locked in a fire proof safe. After project analyses are conducted by Dr. Eichas, all project data will be transferred back to the project leader in Sweden. These data access and safety procedures will be agreed to by all parties (Dr. Eichas and the project leader) involved in a written agreement, prior to any data access by Dr. Eichas.

Etikprövningsmyndigheten
2022-05932-02-325884
2022-11-02

Ethics and data safety in regards SSO and registry data

Virtual classification of geographical data will be performed at Stockholm University in the Department of Psychology and any in person live SSO assessment (if needed) will be conducted on site by trained research team members in the selected geographical areas. No individuals will be studied in the SSO assessment and in cases where people may appear in virtual images, Google street view masks faces, license plates or any other personally identifying images. The SSO assessment will take place according to the SSO data collection protocol and data will be stored electronically in an encrypted cloud service or on password-protected computers in a local, closed computer system at the Department of Psychology, also with data encrypted. Only the researchers working on the study will have access to the files. The SSO method has been used by other researchers for virtual classification of geographical features and the studies thus far have not reported adverse effects of the method (e.g., Bader et al., 2013; Clarke et al., 2010; Odgers et al, 2009, 2012, 2015).

The consent form to parents in Groups 1 and 2 will explain clearly that the purpose of the data collection is at the general and not at the individual child or family level and that neighborhood features (e.g., green spaces, types of residences) of randomly selected areas of participants' postal codes will be observed virtually or in person by the research team as part of the overall project, in order to better understand how neighborhoods maybe important to child and adolescent development and health. Virtual classification of physical conditions in the local environment are observed via digital geographic tools such as Google Earth and the Google Street View (GSV). Virtual classification will be based on the SSO rating form by a trained observer. Participants will also be informed that when results are described actual postal codes will not reported and will be masked and will thus not be identifiable.

Funding and support

This project is funded by FORTE (2019-01661) and Region Stockholm (FoUI-940010).

Key References

Almroth, M. C., Laszlo´, K. D., Kosidou, K., & Galanti, M. R. (2018). Association between adolescents' academic aspirations and expectations and mental health: a one-year follow-up study. *The European Journal of Public Health, 28,* 504–509. doi:10.1093/eurpub/cky025

Anthony, C.J., Elliott, S.N., DiPerna, J.C., & Lei, P-W. (2020a). The SSIS SEL Brief Scales-Student Form: Initial development and validation. *School Psychology, 35(4),* 277-283. doi.org/10.1037/spq0000390

Belfield, C., Bowden, A., Klapp, A., Levin, H., Shand, R., & Zander, S. (2015). The Economic Value of Social and Emotional Learning. *Journal of Benefit-Cost Analysis, 6,* 508-544. doi:10.1017/bca.2015.55

Bierman, K. L., Domitrovich, C. E., Nix, R. L., Gest, S. D., Welsh, J. A., Greenberg, M. T., et al. (2008a). Promoting academic and social-emotional school readiness: the head start REDI program. Child Dev. 79, 1802–1817. doi: 10.1111/j.1467-8624.2008.01227.x

Bierman, K. L., Nix, R. L., Greenberg, M. T., Blair, C., and Domitrovich, C. E. (2008b). Executive functions and school readiness intervention: impact, moderation, and mediation in the head start REDI program. Dev. Psychopathol. 20, 821–843. doi: 10.1017/S0954579408000394

Björnsdotter, A., Enebrink, P., & Ghaderi, A. (2013). Psychometric properties of online administered parental strengths and difficulties questionnaire (SDQ), and normative data based on combined online and paper-and-pencil administration. *Child and Adolescent Psychiatry and Mental Health, 7,* 40. http://www.capmh.com/content/7/1/40

Botvin, G. J., & Griffin, K. W. (2014). Toward the development of preventive interventions to reduce HRSB, HIV/AIDS, and multiple problem behaviors. *Prevention Science, 15,* S81-S83. doi: http://dx.doi.org/10.1007/s11121-014-0461-7

Brickenkamp, R., Schmidt-Atzert, L., and Liepmann, D. (2010). *d2-R.* (Germany: Hogrefe).

Cohen, S., Kamarck, T., & Mermelstein, R. (1983). A global measure of perceived stress. *Journal of Health and Social Behavior, 24,* 386-396.

CPPRG, Conduct Problems Prevention Research Group (1997). School adjustment. Retrieved from the Fast Track Project web site: http://www.fasttrackproject.org/

Criss, M. M., & Shaw, D. S. (2005). Sibling relationships as contexts for delinquency training in low-income families. *Journal of Family Psychology, 19,* 592-600.

Domitrovich, C. E., Cortes, R. C., & Greenberg, M. T. (2007). Improving young children's social and emotional competence: A randomized trial of the preschool "PATHS" curriculum. *The Journal of Primary Prevention, 28,* 67-91. doi: http://dx.doi.org/10.1007/s10935-007-0081-0

Eichas, K., Ferrer-Wreder, L., & Olsson, T. M. (2019). Contributions of positive youth development to intervention science. *Child & Youth Care Forum, 48,* 279-287. doi: http://dx.doi.org/10.1007/s10566-018-09486-1

Elliott, S. N., Anthony, C. J., DiPerna, J. C., Lei, P. W., & Gresham, F. M. (2020a). *SSIS SEL brief scale-parent K-12*. SAIL CoLab.

Elliott, S. N., Anthony, C. J., DiPerna, J. C., Lei, P. W., & Gresham, F. M. (2020b). *SSIS SEL brief scales: User guide and technical manual*. SAIL CoLab.

Elliott, S. N., Anthony, C. J., Lei, P. W., & DiPerna, J. C. (2021). Efficient assessment of the whole social–emotional child: Using parents to rate SEL competencies and concurrent emotional behavior concerns. *School Mental Health.* Published Online First, March 2, 2021. https://doi.org/10.1007/s12310-021-09429-7

Eninger, L., Ferrer-Wreder, L., Eichas, K., Olsson, T. M., Ginner Hau, H., Westling Allodi, M., Smedler, A-C., Sedem, M., Clausen Gull, I., & Herkner, B. (2021). A cluster randomized trial of Promoting Alternative

Etikprövningsmyndigheten
2022-05932-02-325884
2022-11-02

Etikprövningsmyndigheten
2022-05932-02-325884
2022-11-02

Thinking Strategies (PATHS©) with Swedish preschool children. *Frontiers in Psychology, Developmental Psychology, 12,* article: 695288. doi: 10.3389/fpsyg.2021.695288

Ferrer-Wreder, L., Eninger, L., Olsson, T. M., Sedem, M., Allodi Westling, M., & Ginner Hau, H. (in press). The cultural adaptation of interventions to promote positive development: The preschool edition of PATHS in Sweden. In R. Dimitrova & N. Wiium (Eds)., *Handbook of positive youth development in a global context. Research, policy and practice applications*. New York, NY: Springer.

Ferrer-Wreder, L., Eninger, L., Ginner Hau, H., Olsson, T. M., Sedem, M., Thomas, S., & Allodi Westling, M. (2020a). Childcare, Culture, and Child Development: A Swedish Perspective. *The Oxford Handbook of Moral Development: An Interdisciplinary Perspective* (Ed.)., L. A. Jensen. Online Publication Date: Feb 2020, Print Publication Date: Apr 2020. DOI: 10.1093/oxfordhb/9780190676049.013.28

Ferrer-Wreder, L., Bernhard-Oettel, C., Trost, K., Hau, S., & Lindfors, P. (2020b). Exploring lived experiences of parents of youth and youth with a foreign background in Sweden. *Child & Youth Care Forum,* doi:http://dx.doi.org.ezp.sub.su.se/10.1007/s10566-020-09583-0

Fishbein, D. H., Domitrovich, C., Williams, J., Gitukui, S., Guthrie, C., Shapiro, D., & Greenberg, M. (2016). Short-term intervention effects of the PATHS curriculum in young low-income children: Capitalizing on plasticity. *The Journal of Primary Prevention, 37,* 493-511. doi: http://dx.doi.org/10.1007/s10935-016-0452-5

Florean, I. S., Dobrean, A., Păsărelu, C. R., Georgescu, R. D., & Milea, I. (2020). The efficacy of internet-based parenting programs for children and adolescents with behavior problems: A meta-analysis of randomized clinical trials. *Clinical Child and Family Psychology Review, 23*, 510-528. doi: http://dx.doi.org/10.1007/s10567-020-00326-0

Goodman R. (1997). The Strengths and Difficulties Questionnaire: A research note. *Journal of Child Psychology and Psychiatry, 38*, 581-586.

Goodman R (2001) Psychometric properties of the Strengths and Difficulties Questionnaire (SDQ). *Journal of the American Academy of Child and Adolescent Psychiatry*, *40*, 1337-1345.

Hofmann, S. G., & Kashdan, T. B. (2010). The affective style questionnaire: Development and psychometric properties. *Journal of Psychopathology and Behavioral Assessment*, *32*, 255–263. http://dx.doi.org/10.1007/s10862-009-9142-4

Kapetanovic, S., Gurdal, S., Ander, B., & Sorbring, E. (2021). Reported changes in adolescent psychosocial functioning during the COVID-19 outbreak. *Adolescents*, *1*, 10-20. https://doi.org/10.3390/adolescents1010002

Lönn-Rhodin, K., Forster, M., & Enebrink, P. (2015). *Föräldra-Webben/Hempsykologi*.

Malmberg M, Rydell A-M, & Smedje H. (2003). Validity of the Swedish version of the Strengths and Difficulties Questionnaire (SDQ-Swe). *Nordic Journal of Psychiatry, 57*, 357-363.

Odgers, C. L., Caspi, A., et al. (2012). Systematic social observation of children's neighborhoods using Google Street View: a reliable and cost effective method. Journal of Child Psychology and Psychiatry, 53, 1009-1017.

Olsson, T. M., Ferrer-Wreder, L., & Eninger, L. (2014). Advancing school-based interventions through economic analysis. *New Directions for Youth Development, 141,* 117-123.

Parke, R. D. (2004). Development in the family. *Annual Review of Psychology, 55*, 365-399. doi: http://dx.doi.org/10.1146/annurev.psych.55.090902.141528

Pelham, W. E., Gnagy, E. M., Greenslade, B. A., & Milich, R. (1992). Teacher ratings of DSM-III-R symptoms for the Disruptive Behavior Disorders. *Journal of the American Academy of Child and Adolescent Psychiatry, 31*, 210-21.

Ringle, J. L., Mason, W. A., Oats, R. G., & Cogua, J. (2019). Parenting children and adolescents (PARCA) scale English to Spanish translation: An investigation of measurement invariance. *Journal of Family Psychology, 33*(8), 938-944. doi: http://dx.doi.org/10.1037/fam0000559

Robertson, I. H., Manly, T., Andrade, J., Baddeley, B. T., & Yiend, J. (1997). Sustained Attention to Response Task (SART) [Database record]. APA PsycTests. https://doi.org/10.1037/t28308-000

Sasser, T. R., Bierman, K. L., Heinrichs, B., and Nix, R. L. (2017). Preschool intervention can promote sustained growth in the executive-function skills of children exhibiting early deficits. Psychol. Sci. 28, 1719–1730. doi: 10.1177/0956797617711640

Skinner, A. T., & Lansford, J. E. (2020). Experiences Related to COVID-19. Unpublished measure.

Smolker, H. R., Wang, K., Luciana, M., Bjork, J. M., Gonzalez, R., Barch, D. M., . . . Banich, M. T. (2022). The emotional word-emotional face Stroop task in the ABCD study: Psychometric validation and associations with measures of cognition and psychopathology. *Developmental Cognitive Neuroscience*, *53*, 10. doi: http://dx.doi.org/10.1016/j.dcn.2021.101054

Taylor, R. D., Oberle, E., Durlak, J. A., & Weissberg, R. P. (2017). Promoting positive youth development through school-based social and emotional learning interventions: A meta-analysis of follow-up effects. *Child Development, 88,* 1156-1171. doi: http://dx.doi.org.ezp.sub.su.se/10.1111/cdev.12864

Tolan, P., Ross, K., Arkin, N., Godine, N., & Clark, E. (2016). Toward an integrated approach to positive development: Implications for intervention. *Applied Developmental Science, 20,* 214-236. Doi: http://dx.doi.org.ezp.sub.su.se/10.1080/10888691.2016.1146080

Watts, T. W., Gandhi, J., Ibrahim, D. A., Masucci, M. D., & Raver, C. C. (2018). The Chicago school readiness project: Examining the long-term impacts of an early childhood intervention. *PLoS ONE, 13,* 25. doi: http://dx.doi.org/10.1371/journal.pone.0200144

van den Berg, V., Saliasi, E., de Groot, Renate H. M., Chinapaw, M. J. M., & Singh, A. S. (2019). Improving cognitive performance of 9–12 years old children: Just dance? A randomized controlled trial. *Frontiers in Psychology, 10,* 14. doi: http://dx.doi.org/10.3389/fpsyg.2019.00174

Weissberg, R. P., Durlak, J. A., Domitrovich, C. E., and Gullotta, T. P. (2015). "Social and emotional learning: past, present, and future," in Handbook of Social and Emotional Learning: Research and Practice, Eds J. A. Durlak, C. E. Domitrovich, R. P. Weissberg, and T. P. Gullotta (New York, NY: Guilford Press), 3–19.

Wetterborg, D., Enebrink, P. et al. (2019). A pilot randomized controlled trial of internet-delivered parent training for parents of teenagers. *Journal of Family Psychology, 33*, 764-774.

Zigmond, A. S., & Snaith, R. P. (1983). The Hospital Anxiety and Depression Scale. *Acta Psychiatrica Scandinavica, 67,* 361-370. (P8, HAD)

Etikprövningsmyndigheten
2022-05932-02-325884
2022-11-02
