# Supplementary material for: Study protocol for a non-randomized controlled trial of the effects of internet-based parent training as a booster to the preschool edition of PATHS®: Universal edition of the Parent Web
Source: PLoS One. 2023 Apr 27;18(4):e0284926. doi: 10.1371/journal.pone.0284926 (PMC10138844; doi:10.1371/journal.pone.0284926)
Supplement: S3 File — (DOCX) [file pone.0284926.s003.docx]

**Information about the research project to participating parents^[[1]](#footnote-2)^**

We would like to ask you if you would like to participate in a research project called *Children's social and emotional competence and family well-being as children become teenagers*. This project is a continuation of a study your child participated in when s/he was 4 – 5 years old. Stockholm University, which is responsible for the project, collaborates with Karolinska Institute and Statistics Sweden. You are invited to participate in the two sub-studies Follow-up study and Evaluation of the Parent Web. Here you get information about the project and what it means to participate.

If you want the information orally in Swedish or in another language (English, Türkçe, Kurdî, Hrvatski, Bosanski, Srpski), you can contact us by phone at 08-16 38 36. More contact details can be found at the end of this information.

**What kind of project is it and why do you want me to participate?**

*The follow-up study*

When your child was 4–5 years old, he attended a preschool that collaborated with our research group. It then participated in the Swedish preschool version of the PATHS® program. The program is created to develop children's social and emotional competence. Being socially and emotionally competent means that you are good at understanding your own and others' feelings and needs, that you can resolve conflicts and work well together with others. Social and emotional skills develop during childhood. Research shows more and more clearly that social and emotional competence reduces the risk of feeling bad and contributes to children coping better at school and everyday life. It is important to know how we can contribute to a positive development of children's social and emotional competence so that we can support children's development in the best way. More information about the PATHS study and what we found can be found in this mailing.

We know from previous research and from other countries that programs like PATHS, which at an early age focus on strengthening social and emotional competence, can show positive results later in the child's development. Therefore, we want to follow up the children who participated in the program when they went to preschool.

*Evaluation of the parent web*

**Your child is now between 11 and 13 years old. Parenting teenagers can be challenging. As a parent, you may want to know more about adolescence and how you as a parent can support your teenager to reduce the risk of difficulties in the future and reduce conflicts in the family. The parental support programs offered today are mostly aimed at parents with younger children. In addition to following up on how your child is feeling and functioning now, we therefore also want to offer you to participate in a web-based parenting training for teenage parents: the Parent Web program. The program is based on research into risk factors and protective factors for young people's development as well as principles from cognitive behavioral therapy, CBT. The purpose of the program is to strengthen parents in their role through knowledge and tools, so that parents have more energy and the opportunity to help their child in the best way. Understanding the child and the family's strengths can contribute to the family's well-being and functioning.**

**The Parent Web consists of five modules, the first of which is an introduction. There are also six bonus modules covering areas such as friendship & bullying, helping at home, internet, and drugs. Parents can choose to complete the bonus modules that feel important to them. The modules contain both facts and exercises. The content is presented both in text, illustrations, and video-based episodes where researchers are interviewed or where acting parents and teenagers role-play different situations. The content of the program is about how the relationship between parent and child can be strengthened through time together, communication strategies, validation, and positive reinforcement. The program also gives tips to parents on how to deal with conflict, stress, rules, problem solving and agreements. A module takes about an hour to complete and results in homework where parents try out tools and approaches in everyday life.**

**The parents' work is followed by a family guide who acts as a coach and who continuously provides support to the parents via the portal where the program is given. The family guide gives feedback on the work, supports with homework and answers questions. The family guide is supervised by a licensed psychologist. The program takes approximately six to eight weeks to complete. The different parts of the program can be done at times that suit the individual parent.**

**The parent web has proven to be helpful for parents who have children with behavioral problems, and we now want to investigate whether the program is also a good support for parents in families where there are no major problems. The purpose of this part of the project is to investigate whether the Parent Web contributes to improving well-being and relationships within the family.**

*Other parts of the research project*

Another part of our research project is that we investigate what it looks like in the neighborhoods where families participating in the project live. We look at what the physical environment looks like and what resources are available in several randomly selected postcode areas. With this part of the project, we want to investigate whether the physical and social local environment affects the children's well-being and social and emotional competence.

**What does the study mean for you?**

If you choose to participate, we invite you and your child to participate in the Follow-up study and you to participate in the study Evaluation of the parental web.

1. Your child is followed up. We will meet your child at the child's school, or at another place that suits you, at a time that we agree on. The child can then answer questionnaires and solve tasks. The questionnaires are about how your child perceives himself and his life. For example, how the child experiences school, friends, their activity level, and their emotions. The tasks aim to measure abilities that we need to control and direct our thoughts, feelings, and behavior. Answering the questions and tasks takes about an hour.
2. A parent is asked to fill in questionnaires about the family and your child's well-being and functioning. You answer the questionnaires online. These forms are part of the Follow-up Study and we use them to see if the PATHS program has effects after several years. By answering these forms, we also get information about how you are doing in the family before you participate in the parental support program Parent Web.
3. You parents are invited to take part in the internet-based parental support program Parent Web. The program takes six to eight weeks to complete. If you are two parents, either both or just one can take part in the program. The different parts of the program can be done at times that suit you.

We also invite a parent to participate in the study Evaluation of the Parent Web by filling in questionnaires on two occasions. The first opportunity is included when you fill in the forms for the Follow-up Study, point 2 above. Then you get to fill in forms when you have completed the Parents' Web, after approximately 6-8 weeks from the first occasion.

The questions in the form are about your parenting strategies, your child's strengths and difficulties, and the family's way of handling problems and conflicts. You do this via a protected portal on the internet to which you receive a personal password to log in. At any time, you as parents can refrain from continuing to participate in the project and not answer the questions.

We would also like to receive information about what you think about the Parent Network. After you have gone through the various sections of the program, you will therefore also have to answer a few short questions about this.

Participating in the follow-up study therefore takes approximately one hour for your child and approximately one hour for you. Participating in the Follow-up of the Parents Web takes about an additional hour for you. Added to this is the time to implement the parental support program Parent Web.

You can choose which parts of the study you want to participate in. Parents can participate even if the child chooses to decline. The child can participate, with the guardian's approval, even if the parents do not participate.

**What are the risks?**

No risks with the Parent Web or participation in the study have been reported. You can sometimes find it exhausting to answer questions during a study. Therefore, we have tried to shorten the number of questions you have to answer. It takes time from your child's free time to participate in the study. However, most children and young people usually find it fun to do the tasks and talk to an interested adult.

**Are there any benefits?**

There is more and more research showing that parental support programs contribute to children's positive development. The relationship between children and parents is an important factor that contributes to the well-being of family members and the child's development. The Parent Web has been evaluated for parents of teenagers with conflicts at home, with promising results. Previous research shows that Internet-based programs aimed at parents with younger children can have the same beneficial effects as group-based parenting training given on site.

**What happens to my data?**

- The project will collect and register information about you and your child.
- Your answers will be processed so that unauthorized persons cannot access them.

All documents regarding your participation in the project will be treated confidentially. All personnel you come into contact within the project are bound by confidentiality. Exceptions to the duty of confidentiality only apply when a child is so unwell that the project staff is obliged to notify.

The information you provide in the study is entered into a computerized research register. The questionnaires you fill in receive a code number. Your child's information also receives a code number. The researchers have a code key that tells how a code number can be paired with a single individual. Without the code key, information cannot be traced to an individual. The code key is stored separately and locked up. Information included in the research project will only be accessible to authorized researchers.

When results from the study are reported, they will be presented at group level. No individual individuals will be able to be identified. Information that we receive from you will be combined with information from other parents participating in the same parts of the study. Information from your child is combined with the information from the other children participating. We will compare the results for the different groups that are part of different parts of the project. Our overall goal is to investigate whether early general interventions can prevent mental illness and whether the Parents' Network is good for parents and children. By comparing the different groups in the research project, we can better answer those questions.

**How is my personal data handled?**

The data that you and your child provide will be processed according to the EU data protection regulation (which includes GDPR). Stockholm University is the personal data controller for processing the personal data.

According to the EU's data protection regulation, you have the right to receive free information, in the form of a register extract, about your personal data via the university's main registrar registrar@su.se

You also have the right to request that the personal data be corrected, deleted or that the processing of your personal data be restricted. Contact with Stockholm University's data protection officer is via dso@su.se. If you are dissatisfied with how your personal data is processed, you have the right to submit a complaint to the Data Inspectorate, datainspektionen@datainspektionen.se.

**How do I get information about the results of the study?**

The results will be published in the form of scientific reports in international journals. You as a participant can also get information about the results of the study by contacting the project manager in writing.

####

#### Compensation

As compensation for your participation, your child will receive a gift card worth SEK 100 and the parent participating in the study will receive a gift card worth SEK 100 at the beginning and at the end of your participation in the study. It will be a maximum of SEK 300 per family. The gift cards are a small compensation for the time you spend on the project. If one of you chooses to cancel the study, you will still receive the gift cards.

**Voluntary participation**

Participation in the study is completely voluntary. You and/or your child can stop participating at any time without explaining why. You can complete the Parent Web even if you choose to refrain from or cancel participation in the research study.

**Consent**

To participate in the study, you need to give informed consent. Informed consent means that you have received information and understand what it means to participate in the study. As your child is under 15 years of age, the guardian needs to give consent for the child to participate. In order for the child to be able to participate, both the child and guardian need to give consent. Your child has received their own invitation with information about the research project. We will respect your child's decision and your child will also know that they can cancel at any time, without explaining why.

There are two consent forms that you need to read and sign with Bank ID. A form is about consent for the child's participation, and it must be signed by the child's guardian. A form is about a parent's participation in the Follow-up study and in the study Evaluation of the parent web. You can find these forms by scanning the QR code in the letter and following the link to the website.

**Responsible for the study**

*If you would like further information about the study or cancel your participation, contact:*

| Johanna Stålnacke, leg psykolog, Fil. dr,  [johanna.stalnacke@psychology.su.se](mailto:johanna.stalnacke@psychology.su.se)  Telefone: 08- 16 38 28 | Sabina Kapetanovic, Fil. dr, [sabina.kapetanovic@psychology.su.se](mailto:sabina.kapetanovic@psychology.su.se)  Telefone: 08- 16 38 36 |
| --- | --- |

Stockholm University is the research lead for the project.

| Project leader | Laura Ferrer-Wreder, Fil. dr, docent | Psykologiska institutionen  Stockholms universitet  106 91 Stockholm  [laura.ferrer-wreder@psychology.su.se](mailto:laura.ferrer-wreder@psychology.su.se)  Telefon: 08 -16 38 98 |
| --- | --- | --- |
| Responsible researcher  Parent Web | Pia Enebrink,  leg psykolog, Fil dr,  docent | Avdelningen för psykologi  Karolinska institutet  Nobels väg 9-11  171 65 Solna  [pia.enebrink@ki.se](mailto:pia.enebrink@ki.se)  Telefon: [08-524 877 38](tel:08-52487738) |

1. Most often, parents and guardians are the same person. In the research project, we invite the adults who live with and know the child; in this information text we call them "parents". For children under the age of 15 to be able to participate in research, the guardian's permission is required. [↑](#footnote-ref-2)
