# Supplementary material for: Study protocol for a non-randomized controlled trial of the effects of internet-based parent training as a booster to the preschool edition of PATHS®: Universal edition of the Parent Web
Source: PLoS One. 2023 Apr 27;18(4):e0284926. doi: 10.1371/journal.pone.0284926 (PMC10138844; doi:10.1371/journal.pone.0284926)
Supplement: S4 File — (DOCX) [file pone.0284926.s004.docx]

*Children's social and emotional competence and family well-being as children become teenagers.*

**Parental consent to participation in the study**

I have received written information about the research project and the purpose of the partial studies and have been given the opportunity to ask questions and have them answered. I get to keep the written information.

I agree to participate in the Follow-up Study within the project *Children's social and emotional competence and family well-being when children become teenagers*.

I agree to participate in the Evaluation of the Parent Web within the project *Children's social and emotional competence and family well-being when children become teenagers*.

I agree that data about me is processed in the manner described in the information about the research project to participating parents.

|  |  | | |  | |  |
| --- | --- | --- | --- | --- | --- | --- |
|  |  | | |  | |  |
| Signature: |  | | Date: | |  | |
| Name: | |  | | | | |
| Child’s name: | |  | | | | |

| Telephone: | ______________________________________________________________ |
| --- | --- |
| Address:  (Street address and zip code) | ______________________________________________________________ |
|  | ______________________________________________________________ |

| □ | I would like to share research results and give my consent for the research team to contact me |
| --- | --- |
